# A tutorial on selecting, translating, and reporting variance priors in Bayesian multilevel models

**DOI:** 10.3758/s13428-026-03201-y

**Published:** 2026-10-19

**Authors:** Liu Liu

**Affiliations:** School of Social Work, University of Georgia, Athens, GA 30602 USA

**Keywords:** Bayesian multilevel modeling, Variance priors, Scalar variance components, Prior calibration, Prior translation, Small-sample multilevel models

## Abstract

Bayesian multilevel models are increasingly used in psychological, educational, and behavioral research, yet applied guidance on specifying priors for scalar variance components remains limited. This tutorial provides a practical workflow for identifying target variance components, selecting benchmark values, calibrating priors on variance and standard-deviation scales, translating them into software-compatible prior specifications, evaluating their implications through prior predictive checks, posterior comparison, sensitivity analysis, and transparent reporting. We review several commonly used prior families, including gamma, inverse-gamma, half-*t*, and half-Cauchy distributions, and clarify how their parameterizations relate across variance, precision (the reciprocal of variance), and standard-deviation formulations. The tutorial focuses on helping researchers align prior choice with substantive knowledge, model parameterization, software constraints, and sensitivity goals. As a worked example, we use a two-level analysis of TIMSS 2019 data to show how benchmark information from a non-overlapping reference sample can be calibrated into alternative variance-prior specifications and then evaluated in a smaller worked example sample. The empirical illustration is used as a pedagogical example to demonstrate the workflow and its associated checking procedures. We emphasize practical safeguards, including prior-density inspection, prior predictive checking, posterior comparison, convergence assessment, sensitivity analysis, and transparent reporting of prior assumptions. The tutorial concludes by summarizing practical, literature-grounded considerations for documenting prior rationale, implementation decisions, and robustness checks in Bayesian multilevel applications.

**Supplementary Information:**

The online version contains supplementary material available at https://doi.org/10.3758/s13428-026-03201-y.

## Introduction

Multilevel models are widely used to analyze hierarchically structured data in education, psychology, and the behavioral sciences, such as students nested within schools or repeated observations nested within individuals (Luo et al., 2021; McNeish et al., 2017; Snijders & Bosker, 2012). In Bayesian multilevel models, researchers need to specify prior distributions for model parameters, including regression coefficients and variance components such as residual and random-effect variances (Gelman, 2006; Gelman et al., 2014). Although variance priors are often treated as a technical detail, they can substantially affect posterior behavior when the number of higher-level units is limited or when variance parameters are weakly identified (Gelman, 2006; Hox & McNeish, 2020; McNeish, 2016a). In such settings, maximum likelihood estimation may yield unstable variance estimates or boundary estimates, which helps motivate closer attention to how prior distributions regularize variance parameters in Bayesian multilevel models (Browne & Draper, 2000, 2006; McNeish, 2016a, 2016b).

Choosing priors for variance components is often more difficult than choosing priors for fixed effects. Although variance components are defined on a nonnegative scale, the practical issue is not nonnegativity alone. Different prior families can differ in how much mass they allocate near zero, in skewness, and in tail behavior, and these differences can materially affect posterior behavior in weakly identified multilevel settings (Gelman, 2006; Gelman et al., 2014; McNeish, 2016a). Although right-skewness is common for some variance-prior families, it is not a general requirement for priors on variance parameters. Common options for scalar variance priors include gamma and inverse-gamma priors on the variance or precision scale, as well as half-*t* and half-Cauchy priors on the standard deviation scale (Gelman, 2006; Polson & Scott, 2012; van Erp & Browne, 2021). What counts as a “default” prior also varies across software and workflows; some defaults are built into software, whereas others persist through common tutorial examples or legacy practice (Asparouhov & Muthén, 2010; Bürkner, 2017). As a result, applied users may incorrectly assume that default-like choices are neutral or inconsequential, even though prior specification can meaningfully influence posterior summaries in small-sample multilevel settings (Gelman, 2006; McNeish, 2016a, 2016b). The extent of that influence depends on the relative informativeness of the prior and the likelihood and on how closely they align (Gelman et al., 2014; Kruschke, 2014).

This issue is especially important because commonly used variance priors are often described as weakly informative or diffuse even when their behavior can differ substantially across model structures, sample-size conditions, and hyperparameter choices (van Erp & Browne, 2021). Small-hyperparameter inverse-gamma priors, such as Inv-Gamma(0.01, 0.01), can place substantial mass near zero, whereas overly diffuse variance priors can assign substantial probability to implausibly large values; both patterns may affect estimation when the number of clusters is small or variance parameters are weakly identified (Chung et al., 2015; Gelman, 2006; McNeish, 2016a). Existing methodological work has shown that conclusions about variance priors depend on model structure, sample-size conditions, prior family, hyperparameter choice, and evaluation criteria such as bias, root mean square error (RMSE), and credible-interval coverage (Baldwin & Fellingham, 2013; Bolin et al., 2019; McNeish & Stapleton, 2016a; van Erp & Browne, 2021; Zitzmann et al., 2021). For applied researchers, however, this literature can be difficult to translate into concrete modeling decisions, especially when the practical task is to move from a benchmark variance estimate to a defensible prior specification.

This tutorial addresses that practical gap by providing a practical workflow for selecting, translating, implementing, checking, and reporting variance priors for scalar variance components in Bayesian multilevel models. The workflow is designed as a benchmark-informed framework that helps researchers connect plausible variance magnitudes to software-compatible prior specifications while keeping the role of substantive knowledge, model parameterization, software constraints, and sensitivity goals explicit. Other coherent approaches to prior specification, including formal expert elicitation, prior predictive elicitation, hierarchical prior construction, and fully external prior specification, may be more appropriate in some applications. The present tutorial focuses on a benchmark-informed route because applied researchers often need to translate prior studies, historical data, related datasets, or reference-sample information into defensible variance-prior specifications.

We concentrate on commonly used prior families, including gamma, inverse-gamma, half-*t*, and half-Cauchy distributions, and show how these specifications can be related across variance and standard-deviation parameterizations. To illustrate the workflow, we use a brief worked example based on Trends in International Mathematics and Science Study (TIMSS) 2019 data. We present this example as a pedagogical illustration and emphasize practical safeguards such as prior predictive checking, sensitivity analysis, convergence assessment, and transparent reporting of prior assumptions. Throughout, we focus on scalar variance components because they are among the most common priors in applied multilevel modeling and provide a tractable setting for illustrating benchmark-informed calibration across scales. Accordingly, the present tutorial does not attempt to cover priors for covariance matrices of random effects or the broader class of multilevel models in which additional variance and covariance parameters arise. In the present tutorial, the primary concern is with limited numbers of higher-level units rather than cluster size per se, because higher-level variance components are often especially sensitive to estimation difficulties when the number of groups is small, although both features can matter for multilevel estimation in different ways (Maas & Hox, 2005; McNeish & Stapleton, 2016a).

## Overview of common variance prior families

For scalar variance components in Bayesian multilevel models, the most practically relevant prior families can be organized according to the scale on which the prior is imposed. Some priors are specified on the variance or precision scale, such as chi-square, gamma, and inverse-gamma formulations. Others are specified directly on the standard-deviation scale, including half-*t* and half-Cauchy priors, with related alternatives such as half-normal priors also appearing in applied Bayesian workflows (Bürkner, 2017; Gelman, 2006; Gelman et al., 2014). Uniform priors are also sometimes used as bounded weakly informative alternatives, although their behavior depends strongly on the chosen bounds and should therefore not be treated as automatically neutral (Gelman, 2006; Gelman et al., 2014; McNeish, 2016a; Smid & Winter, 2020).

The prior families discussed in this tutorial were selected because they are widely encountered in applied Bayesian multilevel modeling and because, together, they illustrate the main implementation scales that applied researchers must navigate: variance, precision, and standard deviation. Gamma and inverse-gamma priors are useful for explaining variance- and precision-scale formulations, whereas half-*t* and half-Cauchy priors are widely used standard-deviation-scale options in modern Bayesian modeling. Uniform priors are included because they remain familiar bounded alternatives in applied work and provide a useful reminder that simple-looking bounds can carry substantive information. Other prior families, including half-normal priors and penalized-complexity priors, are also important alternatives, especially in modern hierarchical modeling, and the workflow principles developed here, including scale clarification, prior predictive checking, sensitivity analysis, and transparent reporting, can be applied to those specifications as well (Gelman, 2006; Gelman et al., 2014; Simpson et al., 2017).

**Fig. 1:**
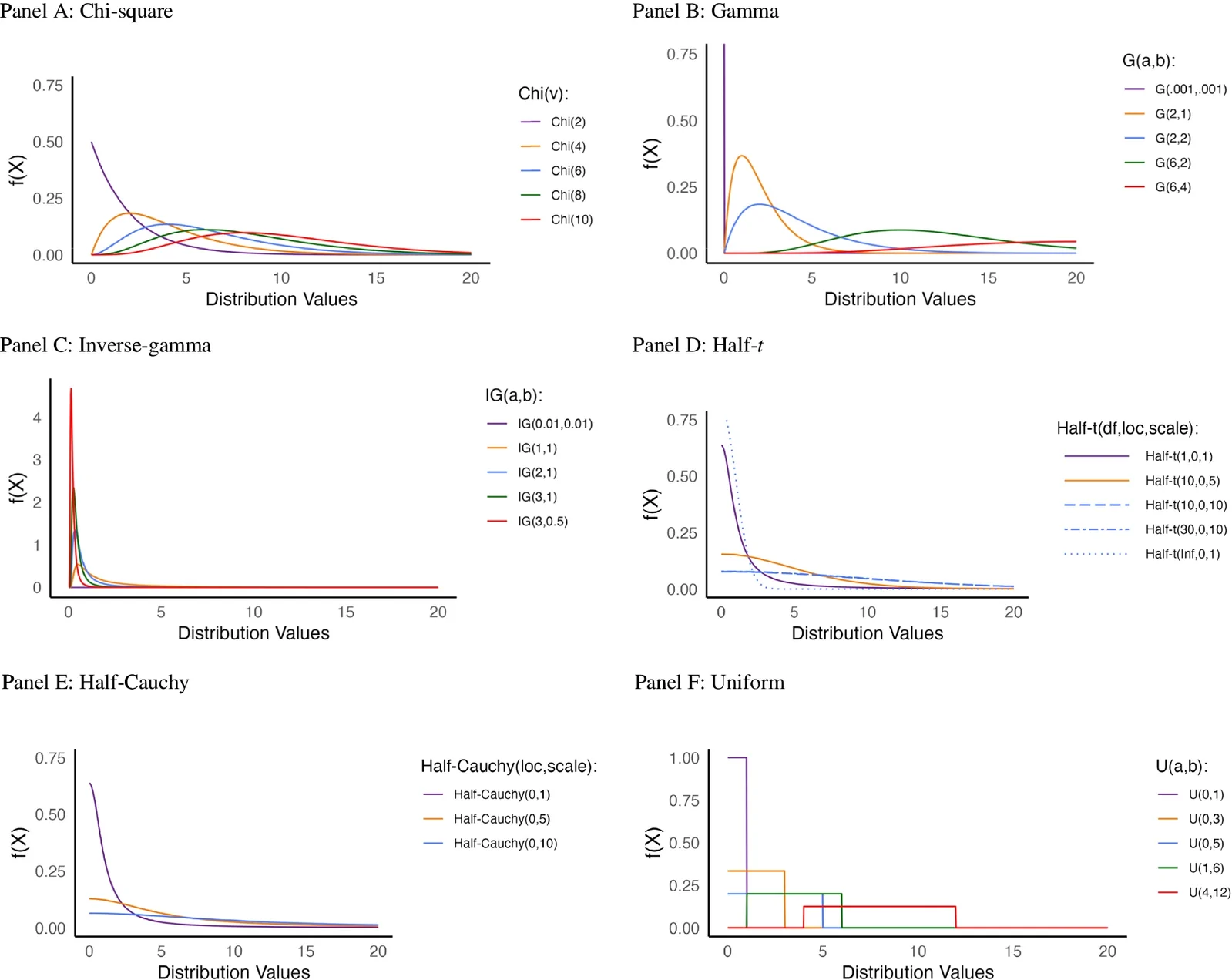
Representative shapes of common prior families for scalar variance components. *Note.* Panels display illustrative examples of the chi-square, gamma, inverse-gamma, half-*t*, half-Cauchy, and uniform families under varying hyperparameter settings. The figure is intended to provide visual intuition about differences in support, concentration near zero, modal location, and tail behavior across prior families commonly used for scalar variance components in Bayesian multilevel models. These examples are shown for descriptive and pedagogical purposes only, rather than to recommend any single family or specification

Because variance parameters can be weakly identified in small-sample multilevel settings, and because prior families differ in how they allocate mass near zero and in the tails, the choice among these families matters both mathematically and substantively (Gelman, 2006; McNeish, 2016a, 2016b). Figure 1 provides a visual comparison of representative shapes from the main prior families discussed in this tutorial. As shown in the figure, these families differ not only in whether they are imposed on the variance, precision, or standard-deviation scale, but also in their concentration near zero, modal location, and tail behavior. These distinctions are consequential in practice because they can shape posterior regularization in meaningfully different ways when the number of higher-level units is limited.

### Chi-square

The chi-square distribution is most useful here as a conceptual bridge rather than as the primary prior used in applied software. Under normal-data theory, sample variances are closely related to scaled chi-square distributions, which makes chi-square-based reasoning a natural starting point for constructing data-informed priors for variance components (Gelman et al., 2014; Mood et al., 1973). Its support is strictly positive, and its shape is governed by degrees of freedom, so it provides an intuitive way to think about both the location of a plausible variance value and the amount of information supporting that value. In practice, chi-square representations are often translated into more convenient prior families, particularly gamma or inverse-gamma formulations.

### Gamma

The gamma distribution is a flexible positive distribution indexed by a shape parameter and either a rate or scale parameter, depending on the parameterization (Gelman et al., 2014; Johnson et al., 1995; Kaplan, 2023). In Bayesian hierarchical modeling, gamma priors are commonly used for variance-related quantities, especially when the model is parameterized in terms of precision. Their flexibility allows researchers to encode different degrees of informativeness: depending on the hyperparameters, a gamma prior can place considerable mass near zero or instead concentrate more strongly around a moderate positive range. This flexibility makes the gamma family useful when researchers wish to anchor a prior to a benchmark estimate while retaining a relatively transparent interpretation of location and spread.

### Inverse-gamma

The inverse-gamma distribution has historically been one of the most common priors for variance parameters because of its conjugate role in normal models and its support on positive values (Forbes et al., 2011; Gelman, 2006; Gelman et al., 2014). However, its practical behavior depends heavily on its hyperparameters. In particular, small-hyperparameter specifications such as Inv-Gamma(0.01, 0.01), which are sometimes described as diffuse or weakly informative, can in fact place substantial mass near zero and may have undesirable consequences for variance estimation in small-sample multilevel settings (Gelman, 2006; McNeish, 2016a). For this reason, inverse-gamma priors should not be treated as default placeholders without examining the implied density and its substantive plausibility.

### Half-t

Half-*t* priors are defined on the standard deviation scale and restrict support to positive values, making them attractive for variance-component modeling when software allows priors to be placed directly on standard deviations (Gelman, 2006; Gelman et al., 2014). Compared with inverse-gamma priors, half-*t* priors are often favored because they can provide regularization near zero while still allowing substantial tail mass for larger standard deviations. Their behavior is controlled by degrees of freedom and scale parameters: smaller degrees of freedom produce heavier tails, whereas larger degrees of freedom produce lighter tails. This makes the half-*t* family especially useful when researchers want a prior that is weakly informative but still responsive to an empirically plausible scale.

### Half-Cauchy

Within the zero-location half-*t* family considered in this tutorial, the half-Cauchy distribution is obtained by setting the degrees of freedom to ν=1 (Gelman, 2006; Polson & Scott, 2012). More generally, both the half-*t* and half-Cauchy priors can be viewed as special cases of the broader folded-noncentral-*t* family. Like other half-*t* priors, it is defined on the positive scale, but it has especially heavy tails, which makes it relatively tolerant of large variance values while still concentrating substantial density near zero. This combination can be appealing when analysts want to avoid the strong near-zero behavior of some inverse-gamma specifications, although the heavy tails also mean that the scale parameter should be chosen thoughtfully rather than by default (Gelman, 2006; Gelman et al., 2014).

### Uniform priors

Uniform priors are sometimes used on the variance or standard-deviation scale because they appear simple and “flat” over a bounded interval. Here, “flat” is used only as an informal descriptive term for constant density over a bounded interval, not as a synonym for the uniform family across prior-specification contexts. However, a bounded uniform prior is not noninformative in any general sense, because its lower and upper limits directly encode substantive assumptions about the parameter’s plausible range. In other words, uniform priors are typically specified by finite lower and upper bounds, and any moments are implied by those bounds rather than chosen directly as prior inputs. In small-sample settings, those bounds can materially affect posterior inferences, especially when the upper bound is chosen arbitrarily large. Accordingly, uniform priors are better understood as bounded weakly informative priors than as neutral defaults (Gelman, 2006; Smid & Winter, 2020).

Because these prior families differ in scale, shape, and tail behavior, they are not interchangeable even when they are all described as “weakly informative.” At the same time, several of these prior families are mathematically related. For example, the chi-square distribution is a special case of the gamma family, inverse-gamma priors can be derived from gamma formulations through reciprocal transformation, and, within the zero-location half-*t* family considered in this tutorial, the half-Cauchy is obtained by setting ν=1 (Koch, 2007; Mood et al., 1973; Polson & Scott, 2012). These relationships are useful because they help researchers organize benchmark information across prior families and software parameterizations while keeping the target parameter scale explicit. However, mathematical relationships across distributions do not imply that the resulting priors will have equivalent practical behavior once combined with the likelihood. Priors aligned on one feature, such as a median, mode, or upper quantile, may still differ in near-zero mass, tail behavior, prior predictive implications, and posterior regularization. The next section builds on these connections and introduces a practical transformation and calibration framework for constructing benchmark-informed variance priors.

## Relationships and prior translation across families

Although these prior families differ in scale, shape, and tail behavior, several of them are mathematically related in ways that are directly useful for prior construction in Bayesian multilevel models. In practice, researchers often begin with a benchmark variance or standard deviation estimate, yet the target prior must ultimately be expressed in the parameterization required by a particular software environment. Accordingly, prior specification is often also a translation problem: researchers may need to move between variance and standard-deviation scales, between variance and precision formulations, or between closely related distribution families while keeping the prior scale substantively interpretable (Bürkner, 2017; Gelman, 2006; Gelman et al., 2014). We do not attempt to provide an exhaustive catalog of all possible distributional transformations. Instead, we focus on relationships that are especially useful for scalar variance components because they clarify how benchmark information can first be expressed conceptually on the variance or precision scale and then translated into the software-compatible forms used in practice. For clarity, gamma distributions are written using the shape-rate parameterization, Gamma $(\alpha , \beta )$, where α is the shape parameter and β is the rate parameter; the equivalent scale parameterization is obtained by setting $\theta =1/\beta$ (Gelman et al., 2014; Johnson et al., 1995).

### Chi-square to gamma

The chi-square distribution is a special case of the gamma family. Specifically, if

$$X\sim {\chi}_{\nu }^{2},$$

then

$$X\sim Gamma\left(\alpha =\frac{\nu }{2}, \beta =\frac{1}{2}\right),$$

under the shape-rate parameterization (Gelman et al., 2014; Mood et al., 1973). More generally, if a variance prior is represented as a scaled chi-square, then it can be written as an equivalent gamma prior with the same support and a directly interpretable location.

This relationship is useful because sample-variance information under normal-data theory is naturally linked to a chi-square distribution. As a result, a benchmark variance estimate can be translated into a gamma prior by choosing degrees of freedom to reflect the amount of information supporting that benchmark and then rescaling the distribution accordingly. For example, if $X\sim {\chi}_{\nu }^{2}$ and

$${\sigma }^{2}=cX,$$

then

$${\sigma }^{2}\sim Gamma\left(\frac{\nu }{2}, \frac{1}{2c}\right).$$

A convenient calibration is to choose $c={s}^{2}/\nu$, where ${s}^{2}$ is a benchmark variance estimate. Under this choice,

$${\sigma }^{2}\sim {\mathrm{Gamma}}\left(\frac{\nu }{2}, \frac{\nu }{2{s}^{2}}\right),$$

which has mean

$$E({\sigma }^{2})={s}^{2}$$

and mode

$$Mode({\sigma }^{2})=\frac{\nu -2}{\nu }{s}^{2}(\nu >2).$$

The practical advantage of this formulation is that it separates two ingredients of prior construction in a transparent way: the benchmark variance ${s}^{2}$, which determines the prior scale, and the degrees of freedom ν, which controls the strength of the prior. Larger values of ν concentrate the prior more tightly around the benchmark, whereas smaller values produce a more diffuse prior. In this sense, the chi-square to gamma connection provides a natural bridge from benchmark variance information to a flexible family that is easy to interpret and straightforward to implement in software that permits gamma-type specifications (Gelman et al., 2014; Johnson et al., 1995).

### Gamma to inverse-gamma

A second useful relationship links gamma and inverse-gamma formulations through reciprocal transformation. If a precision parameter

$$\lambda =\frac{1}{{\sigma }^{2}}$$

is assigned a gamma prior,

$$\lambda \sim \mathrm{G}\mathrm{a}\mathrm{m}\mathrm{m}\mathrm{a}(\alpha , \beta )$$

then the corresponding variance parameter follows an inverse-gamma distribution,

$${\sigma }^{2} \sim \mathrm{I}\mathrm{n}\mathrm{v}-\mathrm{G}\mathrm{a}\mathrm{m}\mathrm{m}\mathrm{a}\left(\alpha , \beta \right)$$

(Forbes et al., 2011; Gelman et al., 2014; Koch, 2007). This equivalence is important because some Bayesian workflows are expressed naturally on the precision scale, whereas others are expressed on the variance scale. The reciprocal relationship makes it possible to translate between these two formulations without changing the underlying positive-support structure of the prior.

At the same time, it is important not to overstate what is preserved by this transformation. A gamma prior on precision and the induced inverse-gamma prior on variance are mathematically linked, but they do not have the same shape on their respective scales. For an inverse-gamma prior on variance,

$$E({\sigma }^{2})=\frac{\beta }{\alpha -1}(\alpha >1),$$

and

$${\mathrm{Mode}}({\sigma }^{2})=\frac{\beta }{\alpha +1},$$

so the practical implications of the prior depend strongly on the hyperparameters and on whether the prior is interpreted on the precision or variance scale (Forbes et al., 2011; Gelman, 2006). This point is especially important in applied multilevel models, where small-hyperparameter inverse-gamma priors are sometimes treated as diffuse defaults even though they can place substantial mass near zero and meaningfully affect variance estimation in small samples (Gelman, 2006; McNeish, 2016a).

For tutorial purposes, the key takeaway is that the gamma to inverse-gamma relationship is most useful when researchers already have a gamma-style formulation, perhaps motivated by chi-square reasoning or by a precision-based model, but need to express the resulting prior on the variance scale. In such cases, the reciprocal transformation provides a principled way to move between formulations while preserving the broader logic of the prior construction. However, the transformed prior should still be examined directly, because equivalent transformations do not guarantee equivalent practical behavior on different parameter scales.

### Half-t to half-Cauchy

A third practically important relationship concerns priors placed directly on the standard deviation scale. For a standard deviation parameter σ, a half-*t* prior can be written as

$$\sigma \sim half-{t}_{\nu }\left(0, A\right), \sigma >0,\nu >0, A>0,$$

where ν denotes the degrees of freedom and A denotes the scale parameter (Gelman, 2006; Gelman et al., 2014). This family is often useful in practice because it allows researchers to control tail behavior through ν while working directly on the standard deviation scale, which is commonly used in Bayesian software such as Stan and the brms package (Bürkner, 2017; Stan Development Team, 2024). A half-normal prior is another possible standard-deviation-scale family, but it is not the family used in the present workflow.

More generally, half distributions and folded distributions are not interchangeable labels in all settings. In the present context, both the half-*t* and half-Cauchy priors can be viewed as special cases of the broader folded-noncentral-*t* family discussed by Gelman (2006). With the zero-location half-*t* family considered here, the half-Cauchy is obtained by setting ν=1:

$$\sigma \sim \mathrm{h}\mathrm{a}\mathrm{l}\mathrm{f}-\mathrm{C}\mathrm{a}\mathrm{u}\mathrm{c}\mathrm{h}\mathrm{y}\left(0, A\right).$$

Thus,

$$\mathrm{h}\mathrm{a}\mathrm{l}\mathrm{f}-\mathrm{C}\mathrm{a}\mathrm{u}\mathrm{c}\mathrm{h}\mathrm{y}\left(0, A\right)\equiv \mathrm{h}\mathrm{a}\mathrm{l}\mathrm{f}-{t}_{1}\left(0, A\right).$$

This relationship is useful for two reasons. First, it shows that the half-Cauchy is not a fundamentally separate prior family, but rather a special heavy-tailed case within the broader half-*t* class. Second, it clarifies why the half-*t* family can serve as a flexible standard-deviation-scale prior family in applied work: by varying ν, researchers can move from heavier-tailed to more concentrated specifications while keeping the same general parameterization. Smaller values of ν imply heavier tails and therefore less resistance to large standard deviations, whereas larger values of ν imply lighter tails and stronger regularization around smaller to moderate values.

From a practical standpoint, the main calibration decision is typically the scale parameter A, which determines the rough magnitude of plausible standard deviations, whereas the degrees of freedom ν govern how readily the prior accommodates larger departures from that scale. For scale parameters such as standard deviations, these priors are typically centered at zero; location-shifted versions are possible, but they require stronger substantive justification and should not be treated as default specifications (Gelman, 2006; Gelman et al., 2014).

In the present tutorial, this relationship is used primarily to support practical prior translation and calibration on the standard deviation scale. Later, in the worked example, half-*t* and half-Cauchy priors are calibrated using a common benchmark on the standard deviation scale so that differences in shape and tail behavior can be compared transparently across prior families.

Taken together, the chi-square to gamma, gamma to inverse-gamma, and half-*t* to half-Cauchy provide the backbone of the transformation workflow used in this tutorial. They are especially useful because they allow researchers to begin with a substantively interpretable benchmark, organize and calibrate that benchmark across common prior families, and then implement the resulting prior in software-specific parameterizations while retaining a clear link between prior scale and substantive meaning. The next section turns these relationships into a practical five-step workflow for choosing, calibrating, implementing, and evaluating variance priors in applied work.

## A practical workflow for prior construction

The relationships described above provide the basis for a practical workflow for constructing variance priors in Bayesian multilevel models. In applied settings, the goal is usually not to identify a universally best prior family, but to translate available information into a prior that is substantively interpretable, compatible with the model parameterization, and transparent about the degree of regularization it imposes (Gelman, 2006; Gelman et al., 2014; McNeish, 2016a). For scalar variance components, this process can be organized into five steps. Table 1 provides a concise roadmap to this workflow, whereas the rationale, formulas, and practical considerations for each step are developed in the text that follows. In practical terms, the workflow moves from identifying the target parameter and implementation scale, to selecting a benchmark and level of prior informativeness, to conceptual calibration on the variance or precision scale, to software-compatible implementation on the scale required by the estimation environment, and finally to prior checking, posterior comparison, and transparent reporting.

**Table 1:** Five-step workflow for constructing variance priors in Bayesian multilevel models

Step	Guiding question	Primary task	Key quantities/formulas	Checks and common pitfalls
1. Identify the target variance components and implementation scale	Which variance component needs a prior, and on what scale must it be specified?	Identify the focal variance component and confirm whether the estimation environment requires a prior on the variance, precision, or standard-deviation scale	The target parameter may be a variance v, a precision λ = 1/v, or a standard deviation $\sqrt{v}$	Check that the prior family matches the software parameterization. A common pitfall is treating priors on variance, precision, and standard deviation as interchangeable
2. Choose benchmark values and a level of prior informativeness	What is a plausible magnitude for this variance component, and how strongly should the prior concentrate around it?	Select a benchmark from external evidence when possible and decide how informative the prior should be	The benchmark may be expressed as ${s}^{2}$ on the variance scale or s on the standard-deviation scale. Prior strength may be indexed by a degrees-of-freedom-like parameter, a shape parameter, or another concentration parameter	Clearly state where the benchmark came from. If it is derived from the same broader dataset, acknowledge that it is not fully independent prior information
3. Calibrate priors on the variance or precision scale	How can the benchmark be expressed as a gamma or inverse-gamma prior?	Use a scaled chi-square representation to calibrate a gamma prior on the variance scale. If the model is parameterized in terms of precision, express the specification as the implied inverse-gamma prior on variance	v~ Gamma (ν/2, $\nu /(2{s}^{2})$), so $E\left(v\right)={s}^{2}$, and Mode $\left(v\right)=((\nu -2)/\nu ){s}^{2}$ for ν>2. If λ ~ Gamma($\alpha , \beta$), then v = 1/λ ~ Inv-Gamma $(\alpha , \beta )$	Check whether the implied prior has a plausible mode, spread, and near-zero behavior. A common pitfall is treating inverse-gamma priors as automatic defaults without inspecting their implications
4. Calibrate priors on the standard-deviation scale	How can the benchmark be expressed as a half-*t* or half-Cauchy prior?	Choose a benchmark standard deviation, select degrees of freedom, and calibrate the scale parameter by matching a prior median or other quantile when the estimation environment requires prior specification on the standard-deviation scale	Half-*t*: $\sqrt{v}$ ~ ${half-t}_{\nu }$(0, A) Half-Cauchy: $\sqrt{v}$ ~ half-Cauchy(0, A), equivalently ${half-t}_{1}(0, A).$ With location 0, the half-Cauchy median equals A	Check whether the implied median and upper quantiles are substantively reasonable. A common pitfall is choosing a scale parameter without checking the tail behavior it implies
5. Inspect the implied prior, assess sensitivity, and report transparently	Does the prior imply plausible values, and are the results robust to reasonable alternatives?	Plot the prior, inspect summary features, conduct prior predictive checks when possible, and refit the model under reasonable alternative prior choices. Report the calibration logic transparently	Useful summaries include the prior mode, median, and upper quantiles. Posterior reporting should also include convergence information such as $\widehat{R}$, effective sample size, and trace-plot behavior when relevant	Check whether conclusions change under alternative but still defensible prior choices. Common pitfalls include reporting only the prior family name, omitting sensitivity analyses, or failing to report convergence diagnostics

The table provides a compact roadmap to the workflow developed in this tutorial for scalar variance components in Bayesian multilevel models. It is intended as an orienting summary rather than a substitute for the prose explanation of each step. The goal is to align benchmark information, parameter scale, prior family, and degree of informativeness in a transparent and substantively interpretable way. In Step 3, gamma distributions are written using the shape-rate parameterization. Thus, if λ ~ Gamma(α, β), where β is a rate parameter, then v = 1/λ follows an inverse-gamma distribution with shape α and scale β

### Step 1. Identify the target variance components and implementation scale

The first step is to identify exactly which variance component is being assigned a prior and on which scale the prior must be specified. This is not merely a software detail: whether the prior is expressed on the variance, precision, or standard-deviation scale can materially affect its implied shape and should therefore be treated as an explicit modeling decision. Depending on the model and software, the relevant target may be a variance, a precision, or a standard deviation. This distinction matters because priors that appear similar conceptually can behave quite differently once expressed on different scales, and software defaults are often tied to a particular parameterization rather than to a general substantive rationale (Bürkner, 2017; Gelman, 2006; McNeish, 2016a). Accordingly, prior construction should begin by clarifying both the model component of interest and the scale required for implementation.

In practical terms, this step answers two questions: (1) What parameter am I placing a prior on, and (2) on what scale must that prior be implemented? Once those questions are answered, the remaining calibration steps become much more transparent.

### Step 2. Choose benchmark values and a level of prior informativeness

The second step is to identify a benchmark value that reflects a plausible magnitude for the target variance component. Ideally, this benchmark should come from external evidence, such as prior studies, historical data, a related dataset, or a clearly separated reference sample. When such information is unavailable, a benchmark derived from a broader or preliminary analysis may still be useful, but it should be interpreted cautiously because it does not constitute fully independent prior information (Gelman, 2008; Gelman et al., 2020).

In practical terms, prior construction requires two inputs: a benchmark location that reflects a plausible magnitude for the target variance component and a choice about prior strength, that is, how strongly the prior should concentrate around that benchmark. Prior strength may be represented through a degrees-of-freedom-like quantity, a shape parameter, or another hyperparameter that governs concentration. Stronger specifications place more mass near the benchmark, whereas weaker specifications allow more dispersion around it.

This step is therefore not only about choosing a plausible value, but also about deciding how confidently that value should constrain the prior. In applied work, these concentration choices should be treated as provisional calibration decisions and checked before fitting the focal model. Analysts can compare a small set of plausible alternatives, such as weaker, moderate, and stronger concentration settings or heavier- and lighter-tailed specifications, and then use prior-density inspection and prior predictive checks to evaluate whether the implied parameter values and outcome variability are substantively reasonable.

### Step 3. Calibrate priors on the variance or precision scale

In plain terms, this step asks how a substantively plausible benchmark variance can be turned into a prior whose location and concentration are both explicit. If the prior is imposed on the variance scale, a convenient starting point is a scaled chi-square representation. It is used here as a convenient pedagogical starting point rather than as a uniquely required one. When prior beliefs are more naturally expressed on the precision scale, or when an inverse-gamma formulation is preferred, the same benchmark information can instead be calibrated directly through the corresponding gamma or inverse-gamma relationships (Forbes et al., 2011; Koch, 2007). Let ${s}^{2}$ denote a benchmark variance and let ν denote the effective amount of prior information. Using the chi-square to gamma relationship, a corresponding gamma prior can be written as

$${\sigma }^{2}\sim {\mathrm{Gamma}}\left(\frac{\nu }{2}, \frac{\nu }{2{s}^{2}}\right),$$

where the gamma distribution is written in shape-rate form. Under this parameterization,

$$E({\sigma }^{2})={s}^{2}$$

and

$$\mathrm{M}\mathrm{o}\mathrm{d}\mathrm{e}({\sigma }^{2})=\frac{\nu -2}{\nu }{s}^{2}\left(\nu >2\right).$$

This formulation is useful because it separates the two main ingredients of prior construction in an interpretable way. The benchmark ${s}^{2}$ determines the overall prior scale, whereas ν determines how tightly the prior is concentrated around that scale. Larger values of ν imply a more informative prior, and smaller values imply a more diffuse prior (Gelman et al., 2014; Johnson et al., 1995).

An alternative starting point arises when the model is parameterized in terms of precision, $\tau =1/{\sigma }^{2}$. In that case, one may assign a gamma prior directly to τ:

$$\tau \sim \mathrm{G}\mathrm{a}\mathrm{m}\mathrm{m}\mathrm{a}\left(\alpha , \beta \right),$$

which induces.

$${\sigma }^{2} \sim$$ Inv-Gamma $$\left(\alpha , \beta \right)$$

through reciprocal transformation (Forbes et al., 2011; Gelman et al., 2014; Koch, 2007). Under this parameterization, the induced inverse-gamma prior on variance has

$$E\left({\sigma }^{2}\right)=\frac{\beta }{\alpha -1},\mathrm{f}\mathrm{o}\mathrm{r} \,\alpha >1$$

and

$$\mathrm{M}\mathrm{o}\mathrm{d}\mathrm{e}({\sigma }^{2})=\frac{\beta }{\alpha +1}.$$

Here, β is interpreted as a rate parameter in the gamma formulation but as a scale parameter in the inverse-gamma formulation. This distinction should be stated explicitly because priors that look symbolically similar may behave differently once expressed on different scales. It also helps explain why inverse-gamma priors should be calibrated and inspected directly rather than treated as automatic defaults, especially in small-sample multilevel settings where their behavior near zero can materially affect posterior summaries (Gelman, 2006; McNeish, 2016a).

#### Illustrative calibration on the variance scale

Suppose a benchmark variance estimate is ${s}^{2}=100$, and suppose ν=20 is judged to reflect a moderate degree of prior information. Then

$${\sigma }^{2} \sim \mathrm{G}\mathrm{a}\mathrm{m}\mathrm{m}\mathrm{a}\left(10, 0.10\right),$$

which has mean 100 and mode 90. Under this calibration, the benchmark value sets the overall prior scale, whereas ν controls how strongly the prior is concentrated around that scale. If the model is parameterized in terms of precision, $\tau =1/{\sigma }^{2}$, a gamma prior on τ induces an inverse-gamma prior on ${\sigma }^{2}$. The two formulations are mathematically linked, but the second hyperparameter is interpreted as a rate in the gamma formulation and as a scale in the inverse-gamma formulation.

For direct comparisons across prior families, researchers may wish to align a common summary feature, such as the prior median or an upper quantile, across specifications. These are not interchangeable calibration targets: matching the median aligns the central location of the prior, whereas matching an upper quantile constrains tail probability and the upper range of values treated as plausible. The appropriate choice therefore depends on the substantive goal of the calibration and should be stated explicitly. In practice, the most interpretable calibration target may also differ by prior family and by parameter scale. For example, mode-based calibration is often convenient for gamma-type priors on the variance scale, whereas median- or quantile-based calibration is often more interpretable for half-*t*-type priors on the standard deviation scale. What matters most is that the chosen calibration target be stated explicitly and linked to a substantively meaningful prior scale. Accordingly, on the variance scale, the benchmark primarily anchors the location of the prior, whereas the concentration parameter governs prior strength; on the precision scale, the corresponding interpretation follows through the induced transformation (Gelman, 2006; Gelman et al., 2014; van de Schoot et al., 2021).

### Step 4. Calibrate priors on the standard-deviation scale

If the software requires a prior on the standard deviation scale, half-*t* priors provide a flexible family for variance components. Let s denote a benchmark standard deviation. A half-*t* prior can be written as

$$\sigma \sim \mathrm{h}\mathrm{a}\mathrm{l}\mathrm{f}-{t}_{\nu }\left(0, A\right), \sigma >0,$$

where ν is the degrees of freedom and A is the scale parameter (Gelman, 2006; Gelman et al., 2014). One practical way to calibrate A is to align a chosen prior quantile with the benchmark value. Because the cumulative distribution function (CDF) of a half-*t* prior satisfies

$${F}_{\mathrm{half} - t}(x)=2{T}_{\nu }(x/A)-1,$$

where ${T}_{\nu }$ denotes the CDF of a standard t distribution with ν degrees of freedom, the p th quantile of the half-*t* distribution is

$${q}_{p}=A{\hspace{0.17em}}{t}_{\nu ,(p+1)/2}.$$

Thus, if researchers want the median of the half-*t* prior to equal a benchmark standard deviation s, they can set

$$A=\frac{s}{{t}_{\nu ,0.75}}.$$

More generally, the half-*t* and half-Cauchy priors can both be viewed as special cases of the broader folded-noncentral-*t* family discussed by Gelman (2006). For the zero-location half-*t* family considered here, the half-Cauchy is obtained by setting ν=1:

$$\sigma \sim \mathrm{h}\mathrm{a}\mathrm{l}\mathrm{f}-\mathrm{C}\mathrm{a}\mathrm{u}\mathrm{c}\mathrm{h}\mathrm{y}\left(0, A\right)\equiv \mathrm{h}\mathrm{a}\mathrm{l}\mathrm{f}- {t}_{1}\left(0, A\right).$$

For the half-Cauchy, the median is exactly A. Thus, if a benchmark standard deviation s is to be used as the median of the prior, the calibration is especially simple:

A=s.

This is one reason that the half-Cauchy is often convenient as a weakly informative prior on the standard deviation scale, although the heavy tails still require careful interpretation (Gelman, 2006; Polson & Scott, 2012).

#### Illustrative calibration on the standard deviation scale

Suppose a benchmark standard deviation is s=10. If a half-Cauchy prior is used and the benchmark is treated as the prior median, then the calibration is

$$\sigma \sim \mathrm{half-Cauchy}(0, 10).$$

If instead a half-t prior with ν=4 is desired and the median is again to be set at 10, then the scale parameter is

$$A=\frac{10}{{t}_{4,0.75}}=\frac{10}{0.741}=13.5.$$

In both cases, the benchmark determines the prior scale, while the prior family and the value of ν control tail behavior and the degree to which larger standard deviations remain plausible.

Thus, on the standard deviation scale, the benchmark anchors the prior scale, whereas the prior family and degrees of freedom govern tail heaviness and regularization.

### Step 5. Inspect the implied prior, assess sensitivity, and report transparently

The final step is to examine what the calibrated priors imply before and after fitting the focal model. This can be done by plotting prior densities, checking summaries such as the mode, median, and upper quantiles, and, when possible, conducting prior predictive checks to determine whether the priors place reasonable probability on plausible outcome variability (Gelman et al., 2014; van de Schoot et al., 2014, 2021). It is also important to assess whether substantive conclusions change meaningfully under alternative but still reasonable prior choices, such as modest changes to benchmark location, prior strength, or prior family (Gelman, 2006; McNeish, 2016a, 2016b; Smid et al., 2020). Once the focal model has been fit using a simulation-based posterior sampler such as Markov chain Monte Carlo (MCMC), transparent reporting should include posterior computation and convergence information, such as the number of chains and iterations, $\widehat{R}$, effective sample size, and visual checks such as trace and autocorrelation plots when relevant. At a minimum, reporting should make clear which prior family was used, on which parameter scale it was imposed, how the hyperparameters were calibrated, what information informed the benchmark, and what sensitivity checks were conducted. Transparent reporting is especially important when defaults are overridden, approximated across parameterizations, or informed by benchmark values derived from related rather than fully external data.

Importantly, these five steps are not specific to any one prior family. Rather, they provide a common framework for translating benchmark information into gamma, inverse-gamma, half-*t*, or half-Cauchy specifications while keeping the calibration logic explicit. Taken together, the five steps provide a practical approach to constructing variance priors that is both technically coherent and substantively interpretable, provided that the benchmark, parameter scale, and degree of prior informativeness are clearly aligned. The worked example in the next section illustrates how this workflow can be implemented in practice using a multilevel analysis of TIMSS 2019 data.

The workflow outlined above is presented at a conceptual level, with emphasis on the mathematical relationships among common prior families and on how benchmark information may be translated across variance, precision, and standard deviation parameterizations. In the worked example that follows, implementation is shaped by the software environment and should therefore be understood as a software-compatible illustration of the broader workflow rather than as a set of exact variance-scale transforms.

## Worked example: TIMSS 2019

To illustrate the tutorial framework in a concrete empirical setting, we used TIMSS 2019 data to show how benchmark information can be translated into software-compatible variance-prior specifications and then evaluated through prior inspection, prior predictive comparison, posterior comparison, and sensitivity analysis. In this worked example, standard-deviation-scale priors were calibrated by matching their median to the benchmark standard deviation. We used median-based calibration because the empirical illustration compared prior families with different skewness and tail behavior on the standard-deviation scale, including half-*t* and half-Cauchy priors whose standard location-0 forms have modes at zero. Under this setup, median matching provided a common and interpretable location target across prior families without requiring shifted-location specifications. Throughout this worked example, the non-overlapping reference sample was used only for benchmark construction, whereas the worked example sample was used for the focal prior comparison and evaluation.

### Step 1. Identify the target variance components and implementation scale

We fit a two-level random-intercept model relating students’ science achievement to mathematics achievement at both the student and school levels. For pedagogical clarity, the worked example used the first plausible values for science and mathematics achievement and treated science achievement as the outcome and mathematics achievement as the predictor. This specification was chosen to provide a familiar and interpretable multilevel illustration rather than to assert a strong causal ordering, although mathematics and science achievement are typically positively associated in TIMSS data (Mullis et al., 2020; Singh et al., 2002). The focal worked example sample consisted of 400 students nested within 40 schools, and a separate non-overlapping reference sample was used later for benchmark construction. This setup was chosen to make the influence of variance-prior specification more visible under a smaller higher-level sample size while keeping the modeling framework familiar to applied researchers.

At Level 1, the predictor was students’ mathematics achievement centered within schools and standardized, so that the student-level effect represented within-school differences in mathematics achievement. At Level 2, the school-level predictor was the school mean of mathematics achievement, standardized across schools, so that it represented between-school differences in the academic environment. This setup corresponds to a manifest within–between decomposition of the mathematics predictor, which is sufficient for the present worked example but does not address latent decomposition approaches, such as the multilevel latent covariate framework proposed by Lüdtke et al. (2008). We also included the cross-level interaction between the student-level and school-level mathematics variables. In substantive terms, the model asked whether science achievement varied as a function of students’ own mathematics achievement, school-average mathematics achievement, and their interaction, while also allowing overall science achievement to vary across schools.

The model can be written as

$${Science}_{ij}={\beta}_{0}+{\beta}_{1}\left({Math}_{ij}^{CWC}\right)+{\beta}_{2}\left({\overline{Math} }_{j}\right)+{\beta}_{3}\left({Math}_{ij}^{CWC}\times {\overline{Math} }_{j}\right)+{u}_{0j}+{\varepsilon}_{ij},$$

where ${Science}_{ij}$ is science achievement for student i in school j, ${Math}_{ij}^{CWC}$ is the cluster-mean-centered student-level mathematics predictor, ${\overline{Math} }_{j}$ is the school-level aggregate mathematics predictor, ${u}_{0j}$ is the school-specific random intercept, and ${\varepsilon}_{ij}$ is the student-level residual. Under the usual normality assumptions, ${u}_{0j}\sim N(0,{\tau}_{00})$ and ${\varepsilon}_{ij}\sim N(0,{\sigma }^{2})$.

Within this model, the two scalar variance components of interest were the between-school random-intercept variance ${\tau}_{00}$ and the within-school residual variance ${\sigma }^{2}$. Because the tutorial focuses on practical prior construction for scalar variance components, these two parameters provided a natural worked example: one higher-level variance component that is often more weakly identified in smaller samples, and one lower-level residual component that is typically more data-dominated.

Although these components can be described conceptually on the variance scale, the model was implemented in the brms package, which parameterizes these quantities on the standard-deviation scale for prior specification. Accordingly, the priors in the worked example were ultimately assigned to the between-school standard deviation, $\sqrt{{\tau}_{00}},$ and the residual standard deviation, σ, rather than directly to ${\tau}_{00}$ or to precision parameters. This distinction is important because it determines how benchmark information must be calibrated into software-compatible prior specifications. 

To ensure that the comparison focused on variance-prior choices, fixed-effect priors were held constant across prior conditions. Specifically, the intercept was assigned a Normal prior centered on the reference-sample outcome mean with standard deviation 100, and the regression coefficients were assigned Normal(0, 50) priors. The intercept prior was chosen to be broad relative to the observed TIMSS score scale while remaining centered on a plausible outcome location for the worked example. Because the predictors were standardized, the coefficient prior was also broad on the TIMSS score scale while avoiding an effectively flat specification. Thus, across prior conditions, the fixed-effect priors remained the same, whereas only the priors on the between-school and residual standard deviations varied across prior families.

The reference-sample centering of the intercept prior served a different role from the benchmark-informed variance priors used in the main comparison. The intercept prior was used as a broad location prior on the TIMSS outcome scale, was not estimated from the worked-example sample, and was held fixed across all prior conditions. It was therefore a background modeling choice rather than a prior-family condition under evaluation. By contrast, the variance-prior specifications were the focal inputs in the tutorial comparison and directly governed the amount of regularization applied to the between-school and residual standard deviations. This distinction clarifies why the prior-likelihood separation issues discussed in the worked example are most consequential for the benchmark-informed variance priors.

All analyses were conducted in R 4.4.1 using the R packages brms 2.22.0 and rstan 2.32.6 on a MacBook Pro with an Apple M3 Max processor and 48 GB unified memory. The worked example was fit using Stan’s Hamiltonian Monte Carlo algorithm with the No-U-Turn Sampler (NUTS), as implemented through the brms interface (Bürkner, 2017). The worked-example models were fit with four chains of 2,000 iterations each, including 1,000 warm-up iterations, with adapt_delta = .99 and max_treedepth = 15. Sensitivity models were fit with stricter sampler settings, using four chains of 4,000 iterations each with 2,000 warm-up iterations, adapt_delta =.995, and max_treedepth = 15, because initial runs indicated the need for more conservative tuning. These settings were used as practical starting values for this model, and adequacy was evaluated from the downstream convergence and sampler diagnostics. 

### Step 2. Choose benchmark values and a level of prior informativeness

The next step was to select benchmark values for the target variance components and decide how strongly the priors should be concentrated around them. In principle, benchmark information may come from prior studies, historical data, or other external evidence. In this worked example, we used a non-overlapping reference sample drawn from the broader TIMSS 2019 dataset while reserving a separate smaller-sample dataset for the focal prior comparison. This design provided an empirically grounded basis for benchmark construction while avoiding direct reuse of the same observations for both benchmark estimation and worked example model fitting.

Specifically, the reference sample consisted of 7,408 students nested within 233 schools, whereas the worked example sample consisted of 400 students nested within 40 schools. The same two-level random-intercept model described in Step 1 was fit to the reference sample using maximum likelihood, and the resulting variance-component estimates were used as benchmark values for prior calibration. Fitting the model to the reference sample yielded a benchmark between-school random-intercept standard deviation of 12.20 and a benchmark residual standard deviation of 46.08. On the variance scale, these corresponded to 148.77 and 2,123.38, respectively. These benchmark values served as empirical location targets for the subsequent prior specifications. Because these benchmark values were obtained from a reference-sample maximum-likelihood fit, their usefulness depends on how well that reference-sample estimate reflects the target variance structure. If the benchmark estimate is poor, biased, or inflated, the resulting benchmark-informed priors may pull posterior inferences in a correspondingly misleading direction. Accordingly, the reference-sample estimate should be treated as an empirical input for calibration rather than as a ground-truth value.

The use of a non-overlapping reference sample avoids direct reuse of the same observations for both benchmark estimation and focal model fitting, but it does not guarantee that the reference sample and worked example sample share the same variance structure. If the target sample differs meaningfully from the reference sample, benchmark-informed priors may stabilize estimation at the cost of shrinkage toward a misaligned value. For this reason, benchmark construction should be treated as a substantive assumption rather than as a neutral preprocessing step, and in applied settings benchmark information may be triangulated from prior studies, related datasets, expert elicitation, or data-splitting strategies when appropriate. When multiple sources of benchmark information are available, researchers should prioritize the most substantively comparable external evidence, examine whether the source and target samples are likely to share a similar variance structure, and treat disagreement across sources as a reason to calibrate and compare a range of plausible priors rather than a single benchmark (Garthwaite et al., 2005; Williams et al., 2021).

At this stage, it is important to distinguish benchmark location from prior informativeness. The benchmark values identify plausible magnitudes for the two variance components, but they do not by themselves determine how tightly the prior should be concentrated around those values. Accordingly, the worked example treated benchmark location, prior concentration, and tail behavior as related but distinct calibration decisions. The benchmark values anchored the location of the prior specifications, whereas shape parameters, degrees-of-freedom parameters, and scale settings governed how much dispersion or tail weight remained around those benchmark values.

In practice, these calibration settings should be chosen through an explicit calibration-and-checking process. One practical strategy is to define a small set of plausible alternatives, such as weaker, moderate, and stronger concentration settings for shape-based priors, or heavier- and lighter-tailed specifications for half-*t* priors, and then examine their implications through prior-density plots and prior predictive checks. If the prior predictive distribution assigns substantial probability to outcome patterns that are substantively implausible, a more concentrated prior, a lighter-tailed specification, or a different calibration target may be more appropriate. If the prior predictive distribution is unrealistically narrow, a weaker or heavier-tailed prior may better reflect uncertainty. These choices should be reported as analyst-specified calibration decisions and evaluated through sensitivity analyses.

For the main worked example, we chose a moderately concentrated setting for the between-school standard deviation and a more concentrated setting for the residual standard deviation. This asymmetry was intentional. Higher-level variance components are often less stable and less directly identified in smaller multilevel samples than lower-level residual variation, especially when the number of higher-level clusters is limited (Bolin et al., 2019; Hox & McNeish, 2020; McNeish & Stapleton, 2016a). Therefore, the worked example allowed greater prior dispersion for the between-school component while using a tighter calibration for the residual component. In addition, the half-*t* family was represented using a moderately heavy-tailed setting so that its behavior could be contrasted with both the more concentrated gamma-family specifications and the heavier-tailed half-Cauchy alternative (Gelman, 2006; Polson & Scott, 2012). At this stage, the emphasis is on the logic of the informativeness choice; the exact family-specific calibrations are introduced in Steps 3 and 4.

We also included a baseline reference condition that was not benchmark-calibrated in the same way as the four focal prior families. Specifically, the baseline condition used a user-defined weakly informative half-*t*(3, 0, 20) specification on the standard-deviation scale for both variance components. This baseline condition served as a less structured comparison point against which the benchmark-informed prior families could be evaluated.

### Step 3. Calibrate priors on the variance or precision scale

Having established benchmark values and an overall strategy for prior informativeness, we next expressed the benchmark information conceptually on the variance or precision scale before turning to software-compatible implementation. This step is useful because many prior formulations for variance components are introduced and interpreted on the variance scale, even when final implementation occurs on the standard-deviation scale. In the present tutorial, this intermediate step serves as a conceptual bridge between the general relationships reviewed earlier and the standard-deviation-scale priors introduced in Step 4. It is also pedagogically useful because benchmark information is often easier to interpret on the variance scale, and many commonly discussed prior relationships are first presented in that parameterization, even when final implementation occurs elsewhere.

A convenient starting point is a gamma prior on the variance scale. Using the shape-rate parameterization, an illustrative variance-scale prior can be written as

$${\sigma }^{2}\sim {\mathrm{Gamma}}\left(\frac{\nu }{2}, \frac{\nu }{2{s}^{2}}\right),$$

where ${s}^{2}$ denotes the benchmark variance and ν is a concentration-like tuning parameter. Under this parameterization, the prior mean is ${s}^{2}$, and the prior mode is $\frac{\nu -2}{\nu }{s}^{2}$ for ν>2. This formulation separates the two main ingredients of prior construction in a transparent way: the benchmark variance determines prior location, whereas ν determines prior concentration around that location.

In the TIMSS worked example, the benchmark variances obtained from the non-overlapping reference sample were 148.77 for the between-school random intercept and 2,123.38 for the residual component. Table 2 summarizes the corresponding illustrative variance-scale calibrations. For the between-school variance, we used ν=20, which yields

$${\sigma}_{\mathrm{between}}^{2}\sim {\mathrm{Gamma}}(10.0, 0.0672),$$

and for the residual variance, we used ν=50, which yields

**Table 2:** Illustrative variance-scale gamma calibrations for the TIMSS worked example

Variance component	Benchmark variance ${s}^{2}$	Illustrative concentration ν	Shape $\alpha =\nu /2$	Rate $\beta =\nu /(2{s}^{2})$	Variance-scale gamma prior
Between-school random intercept	148.77	20	10.0	0.0672	Gamma(10.0, 0.0672)
Residual	2,123.38	50	25.0	0.0118	Gamma(25.0, 0.0118)

Benchmark variances were obtained from the non-overlapping reference sample using the same two-level random-intercept model described in Step 1. The benchmark variances are the squared benchmark standard deviations reported in Step 2. The concentration parameter ν was chosen illustratively to represent a more moderate setting for the between-school variance and a stronger setting for the residual variance; it was not estimated from the data. Using the shape-rate parameterization, the illustrative variance-scale prior was defined as ${\sigma }^{2}\sim Gamma(\nu /2,\nu /(2{s}^{2}))$. Thus, for the between-school variance, ${s}^{2}=148.77$ and ν=20 yield α=10.0 and β=0.0672; for the residual variance, ${s}^{2}=\mathrm{2,123.38}$ and ν=50 yield α=25.0 and β=0.0118. These are conceptual variance-scale calibrations used to clarify the workflow, not the final priors used for model fitting in brms

$${\sigma}_{\mathrm{residual}}^{2}\sim {\mathrm{Gamma}}(25.0, 0.0118).$$

These settings were chosen to be consistent with the general strategy introduced in Step 2: a moderate concentration setting for the between-school variance and a stronger concentration setting for the residual variance. For example, for the between-school variance, substituting ${s}^{2}=148.77$ and ν=20 into the gamma calibration yields $\alpha =\nu /2=10.0$ and $\beta =\nu /(2{s}^{2})=20/(2\times 148.77)=0.0672$. This worked calculation is included to make the variance-scale calibration transparent before turning to the software-compatible standard-deviation-scale implementation.

Here, ν functions as an illustrative tuning parameter rather than a data-estimated quantity. Its role is to control prior concentration while keeping benchmark location fixed. In the present worked example, ν=20 was used to represent a moderate variance-scale concentration setting for the between-school component, whereas ν=50 was used to represent a stronger setting for the residual component. These values were selected to make the calibration concrete and interpretable in the tutorial, not to define uniquely correct hyperparameters. Their implications were therefore examined later through sensitivity analyses.

This variance-scale representation also clarifies the relationship between variance-based and precision-based formulations. Because precision is the reciprocal of variance, a gamma prior on precision implies an inverse-gamma prior on variance under reciprocal transformation. Thus, the same benchmark information can be expressed on either the variance scale or the precision scale, depending on the model parameterization and software environment. In the present worked example, however, these variance-scale calibrations are used primarily for conceptual clarification rather than for final estimation.

Accordingly, the priors shown in Table 2 should be interpreted as illustrative variance-scale calibrations that connect the empirical benchmark values to the broader prior-construction workflow. They are not the final priors used for model fitting in brms. Instead, they show how benchmark information and an explicitly chosen level of prior concentration can first be expressed in a familiar variance-scale form before being translated into software-compatible standard-deviation-scale priors in the next step.

### Step 4. Calibrate priors on the standard-deviation scale

The next step was to express the benchmark information as standard-deviation-scale priors suitable for software implementation. This step is especially important in the present worked example because the model was fit in brms, where priors for group-level standard deviations and the residual standard deviation are specified directly on the standard-deviation scale, not on the variance or precision scale. Thus, although Step 3 expressed the benchmark information in a conceptually useful variance-scale form, the fitted priors required a separate software-compatible calibration on the standard-deviation scale.

#### Common calibration target

A central issue in this step is the choice of a common calibration target across prior families. In the present tutorial, all four focal prior families were calibrated so that their median matched the corresponding benchmark standard deviation. Median matching is used here as a location-based calibration rule; upper-quantile matching would serve a different purpose by calibrating tail probability and the upper range of values treated as plausible. This median-based calibration was used for two reasons. First, it provides a common and interpretable location target across prior families with different skewness and tail behavior. Second, it is especially useful on the standard-deviation scale because some prior families commonly used in practice, particularly half-*t* and half-Cauchy priors with location 0, have their mode at zero. As a result, mode-based alignment would not yield a practically comparable family-wise calibration across all four prior families.

At the same time, matching the median does not make the priors practically equivalent. It aligns only one summary feature of each prior. The calibrated priors may still differ in near-zero mass, tail behavior, induced prior predictive variability, and posterior regularization once combined with the likelihood. For this reason, median matching should be understood as a transparent calibration device for organizing the comparison, not as a claim that the prior families preserve equivalent informativeness across parameterizations.

#### Data-informed versus analyst-specified inputs

At this stage, it is also important to distinguish data-informed benchmark values from analyst-specified calibration settings. The benchmark standard deviations were obtained from the non-overlapping reference sample and therefore served as empirical location targets for prior calibration. In contrast, the shape parameters for the gamma and inverse-gamma priors and the degrees-of-freedom parameter for the half-*t* prior were specified by the analyst to represent different levels of prior concentration and tail behavior. In the present worked example, shape = 12 was used to represent a moderate concentration setting for the between-school standard deviation, shape = 30 was used to represent a stronger concentration setting for the residual standard deviation, and *df* = 4 was used to represent a moderately heavy-tailed half-*t* prior. These values were chosen to produce a substantively interpretable and pedagogically clear comparison across prior families.

In applied analyses, analogous calibration settings should be selected by examining the substantive plausibility of the resulting prior distribution and by checking the implied prior predictive distribution on the outcome scale. For example, analysts may compare weaker, moderate, and stronger concentration settings, or heavier- and lighter-tailed specifications, before selecting the main prior condition. The present worked example follows this logic by using the main shape and degrees-of-freedom values as illustrative calibration choices and then evaluating their implications through prior-density inspection, prior predictive comparison, and sensitivity analyses.

#### Family-specific standard-deviation-scale calibrations

Using this strategy, the focal prior comparison in the worked example was carried out on the standard-deviation scale for the two target parameters identified in Step 1: the between-school random-intercept standard deviation and the within-school residual standard deviation. The benchmark standard deviations obtained from the non-overlapping reference sample were 12.20 and 46.08, respectively. Table 3 summarizes the resulting standard-deviation-scale calibrations for the four focal prior families: gamma, inverse-gamma, half-*t*, and half-Cauchy. These calibrations apply only to the priors on the between-school and residual standard deviations; the fixed-effect priors were held constant across prior conditions. For the between-school standard deviation, the calibrated priors were Gamma(shape = 12, rate = 0.9566), inverse-gamma(shape = 12, scale = 142.32), half-*t*(*df* = 4, scale = 16.47), and half-Cauchy(scale = 12.20). For the residual standard deviation, the corresponding calibrations were Gamma(shape = 30, rate = 0.6438), inverse-gamma(shape = 30, scale = 1367.08), half-*t*(*df* = 4, scale = 62.21), and half-Cauchy(scale = 46.08).

**Table 3:** Standard-deviation-scale prior calibrations for the TIMSS worked example

Variance component	Benchmark SD	Prior family	Fixed setting	Calibration rule	Resulting hyperparameters
Between-school random intercept SD	12.20	Gamma	shape = 12	Rate chosen so that prior median = benchmark SD	shape = 12, rate = 0.9566
	12.20	Inverse-gamma	shape = 12	Scale chosen so that prior median = benchmark SD	shape = 12, scale = 142.32
	12.20	Half-*t*	*df* = 4	Scale chosen so that prior median = benchmark SD	*df* = 4, scale = 16.47
	12.20	Half-Cauchy	none	Scale set equal to benchmark SD	scale = 12.20
Residual SD	46.08	Gamma	shape = 30	Rate chosen so that prior median = benchmark SD	shape = 30, rate = 0.6438
	46.08	Inverse-gamma	shape = 30	Scale chosen so that prior median = benchmark SD	shape = 30, scale = 1367.08
	46.08	Half-*t*	*df* = 4	Scale chosen so that prior median = benchmark SD	*df* = 4, scale = 62.21
	46.08	Half-Cauchy	none	Scale set equal to benchmark SD	scale = 46.08

Benchmark standard deviations were obtained from the non-overlapping reference sample using the same two-level random-intercept model described in Step 1. All four prior families were calibrated on the standard-deviation scale so that their median matched the corresponding benchmark SD. For gamma priors, the shape parameter was fixed and the rate parameter was solved numerically so that the prior median equaled the benchmark SD. For inverse-gamma priors, the shape parameter was fixed and the scale parameter was solved numerically so that the prior median equaled the benchmark SD. For half-*t* priors, degrees of freedom were fixed at 4 and the scale parameter was chosen using $A=s/{t}_{\nu ,0.75}$, where s denotes the benchmark SD. For the half-Cauchy prior, which is the ν=1 special case of the half-*t* family, the median equals the scale parameter, so the scale was set directly equal to the benchmark SD. The baseline reference condition is not shown because it was not benchmark-calibrated in the same way as the four focal prior families

For example, consider the gamma prior for the between-school random-intercept standard deviation. The benchmark standard deviation from the non-overlapping reference sample was 12.20. To construct a software-compatible gamma prior on the standard-deviation scale, we fixed the shape parameter at 12 and then solved for the rate parameter so that the prior median equaled the benchmark value. Under this calibration, the resulting prior was Gamma(12, 0.9566). The same logic was applied to the other prior families, although the parameter solved for differed by family. For inverse-gamma priors, the shape parameter was fixed and the scale parameter was chosen so that the prior median matched the benchmark standard deviation. For half-*t* priors, the degrees of freedom were fixed at 4 and the scale parameter was chosen so that the prior median matched the benchmark standard deviation. For the half-Cauchy prior, the scale parameter was set equal to the benchmark standard deviation because, under this parameterization, the prior median equals the scale.

**Fig. 2:**
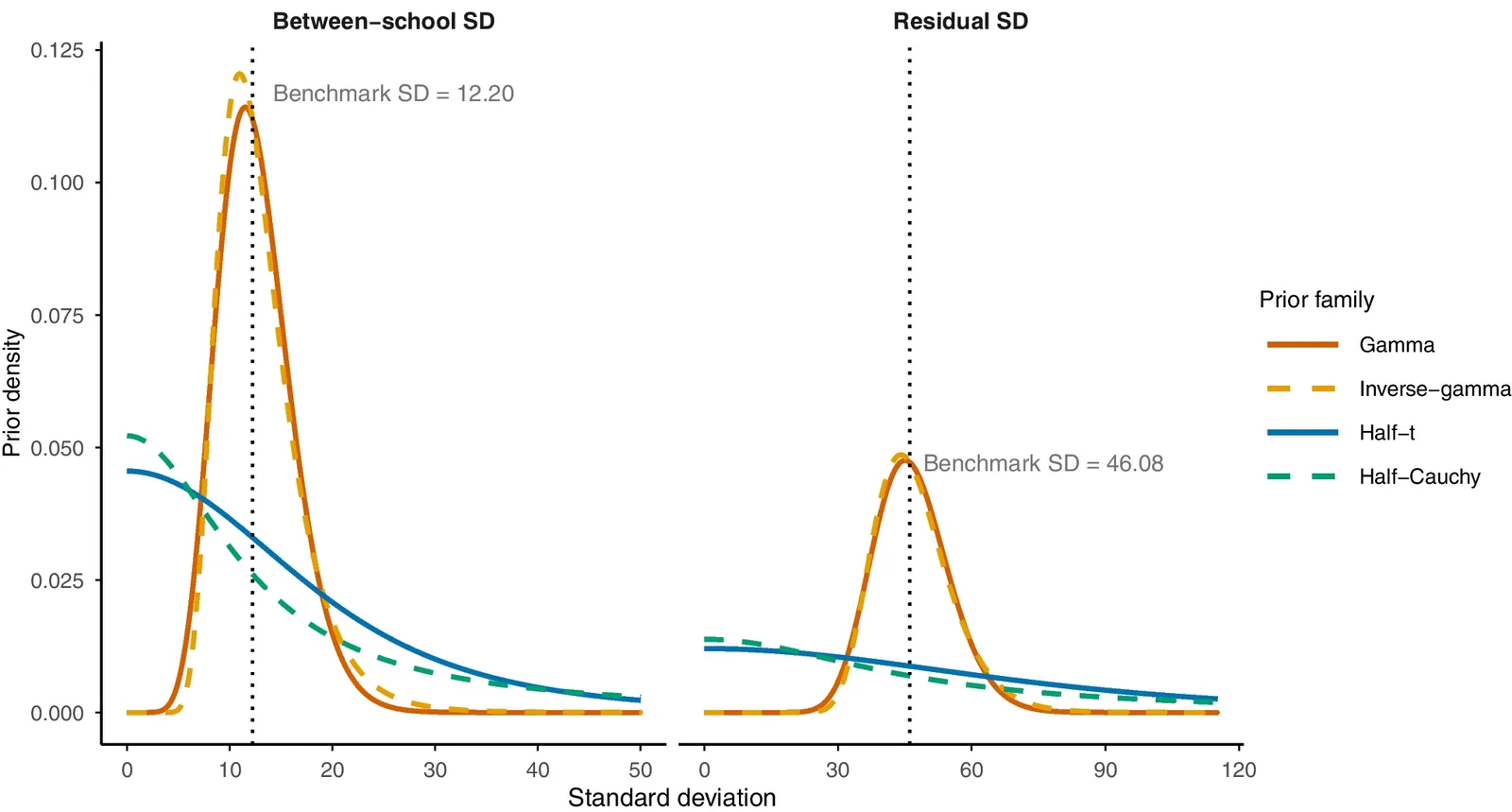
Prior densities for calibrated variance-prior families on the standard-deviation scale. *Note.* The left panel shows priors for the between-school random-intercept standard deviation, and the right panel shows priors for the residual standard deviation. Dotted vertical lines indicate benchmark standard deviations estimated from the non-overlapping reference sample. All prior families were calibrated so that their median matched the corresponding benchmark standard deviation. Gamma and inverse-gamma priors are shown as software-compatible standard-deviation scale calibrations for implementation in brms, rather than as exact variance-scale transformations

Figure 2 shows the prior-density comparison on the standard-deviation scale. Even when all prior families are aligned to the same benchmark median, they differ meaningfully in shape and tail behavior. The gamma and inverse-gamma priors place relatively more mass near the benchmark values, whereas the half-*t* and especially the half-Cauchy priors retain heavier tails and therefore assign greater prior probability to larger standard deviations. This distinction is important in practice because prior families that share a common benchmark location can still differ in how strongly they regularize the posterior, particularly when information about higher-level variance components is limited.

These comparisons also illustrate the difference between mathematical translation and practical calibration. Distributional relationships across variance, precision, and standard-deviation scales are useful for organizing prior construction, but they do not guarantee that priors will have equivalent practical behavior in a fitted multilevel model. A calibrated prior must therefore be evaluated not only by its formula or matched summary value, but also by its implied density, prior predictive distribution, posterior behavior, and sensitivity to reasonable alternative specifications.

In addition to the four benchmark-informed prior-family conditions, we also retained a baseline reference condition for comparison. As described in Step 2, this baseline specification used a user-defined weakly informative half-*t*(3, 0, 20) prior on the standard-deviation scale for both variance components. The purpose of this baseline condition was not to represent a benchmark-calibrated family, but to provide a less structured reference point against which the four calibrated prior families could be evaluated.

Taken together, Step 4 completes the transition from benchmark identification to software-ready prior specification. It shows how a common benchmark can be used to organize multiple prior-family calibrations on the standard-deviation scale while preserving meaningful differences in concentration and tail behavior. The resulting calibrated priors then provide the basis for the prior inspection, prior predictive comparison, posterior comparison, and sensitivity analyses reported in the next step.

### Step 5. Inspect the implied prior, assess sensitivity, and report transparently

With the priors calibrated, the final step was to examine what they implied before and after fitting the focal model, assess whether substantive conclusions were sensitive to reasonable alternative specifications, and report those choices transparently. In a tutorial context, this step is essential because prior construction does not end once hyperparameters have been specified. Even a mathematically valid prior may be a poor practical choice if it implies implausible outcome variation, places too much mass in unrealistic regions of the parameter space, or yields posterior behavior that is overly sensitive to arbitrary calibration settings. Accordingly, the present worked example evaluated the calibrated priors in four complementary ways: prior inspection, prior predictive checking, posterior comparison, and sensitivity and diagnostic assessment.

We began by inspecting the priors on the parameter scale itself. As shown in Fig. 2, the four benchmark-informed prior families were aligned to a common median on the standard-deviation scale, but they differed meaningfully in shape and tail behavior. The gamma and inverse-gamma priors concentrated relatively more mass near the benchmark values, whereas the half-*t* and especially the half-Cauchy priors retained heavier tails and therefore allowed larger standard deviations to remain more plausible a priori. This inspection established that the calibrations were not interchangeable, even though they were aligned to the same benchmark location.

**Fig. 3:**
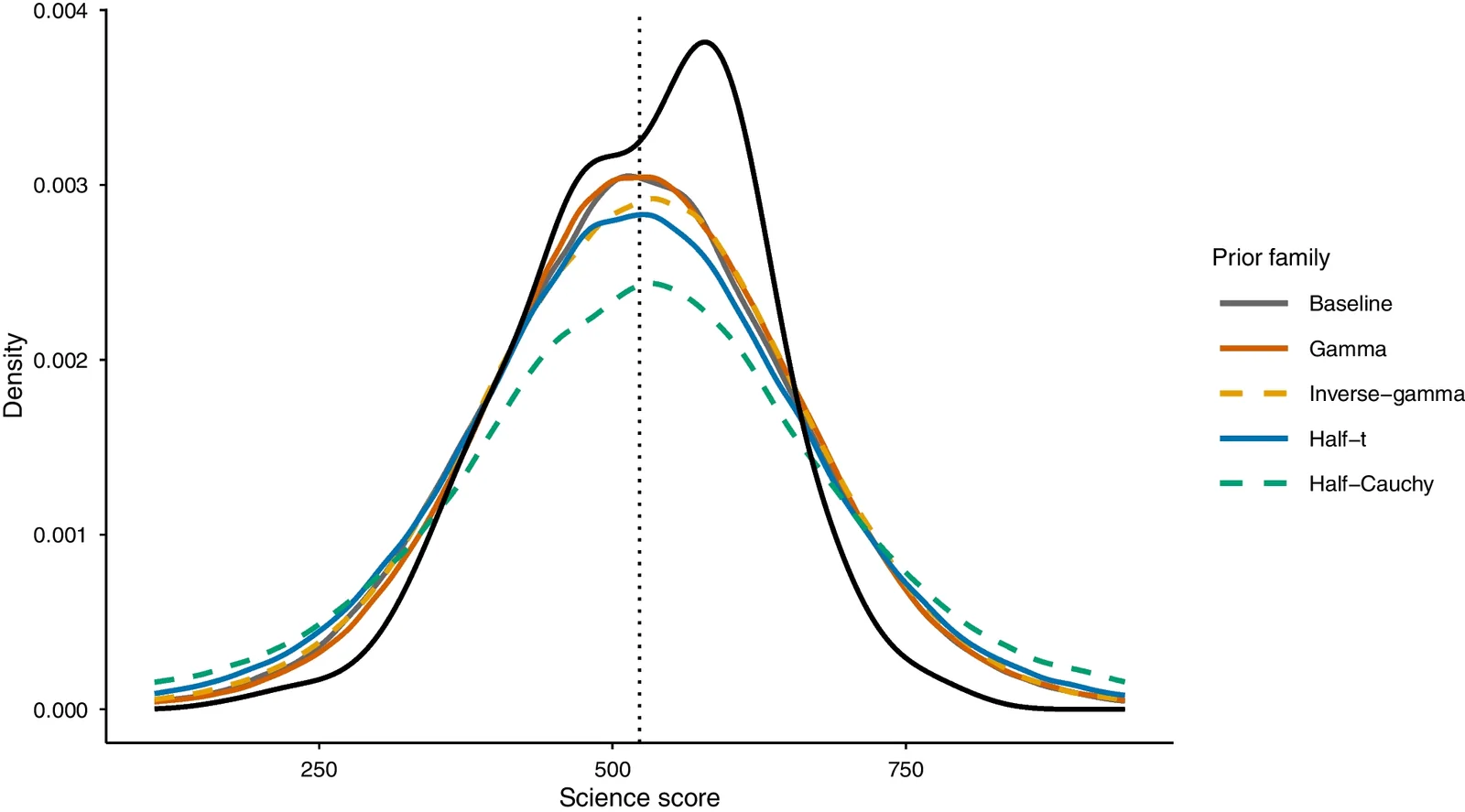
Prior predictive distributions for science achievement across prior conditions in the TIMSS worked example. *Note.* The black curve represents the observed distribution of science achievement in the worked example sample, and the colored curves represent prior predictive distributions under each prior condition. The vertical dotted line marks the observed mean of science achievement in the worked example sample. For visual clarity, the x-axis was restricted to the observed mean ± four observed standard deviations. Across conditions, fixed-effect priors were held constant, so differences in the prior predictive distributions primarily reflect the variance-prior specifications

We then examined the implications of these priors on the outcome scale through prior predictive checks. Figure 3 shows the prior predictive distributions for science achievement under the alternative prior conditions. Across conditions, the prior predictive distributions were centered in a broadly similar region of the observed outcome scale, indicating that the benchmark-informed calibrations were aligned to a common location target. At the same time, the heavier-tailed specifications implied greater dispersion, particularly for the half-Cauchy prior. This pattern is also reflected in Table 4, which summarizes the prior predictive means and standard deviations under each condition. For example, the median prior predictive standard deviation was smaller under the baseline and gamma conditions than under the half-*t* and half-Cauchy conditions, with the half-Cauchy prior showing the broadest upper range. These comparisons show that differences among prior families are not merely mathematical. They also affect the amount of variability implied for the observed outcome before fitting the model. In this sense, prior predictive checking complements the parameter-scale comparison in Fig. 2 by showing how differences among prior families translate into substantively interpretable implications on the observed-data scale.

**Table 4:** Prior predictive summary statistics for science achievement in the TIMSS worked example

Prior condition	Observed mean	Observed SD	Prior predictive mean, median	Prior predictive mean, 95% interval	Prior predictive SD, median	Prior predictive SD, 95% interval
Baseline	523.19	103.34	520.34	[334.24, 723.71]	81.43	[31.73, 161.99]
Gamma	523.19	103.34	527.44	[338.36, 696.89]	91.39	[51.31, 181.12]
Inverse-gamma	523.19	103.34	526.23	[325.71, 735.51]	88.90	[51.17, 144.46]
Half-*t*	523.19	103.34	527.56	[337.28, 688.35]	102.55	[47.50, 202.67]
Half-Cauchy	523.19	103.34	525.12	[319.38, 717.30]	117.11	[41.62, 625.19]

Observed statistics refer to the distribution of science achievement in the worked example sample. Prior predictive summaries were obtained from prior-only simulation under each prior condition. “Prior predictive mean, median” denotes the median of the simulated outcome means across prior-predictive draws, and “prior predictive SD, median” denotes the median of the simulated outcome standard deviations across draws. The reported 95% intervals are equal-tailed prior predictive intervals for these simulated summary statistics. The baseline condition used a user-defined weakly informative half-*t*(3, 0, 20) prior on the standard-deviation scale and is included as a reference condition rather than as a benchmark-calibrated prior family

**Fig. 4:**
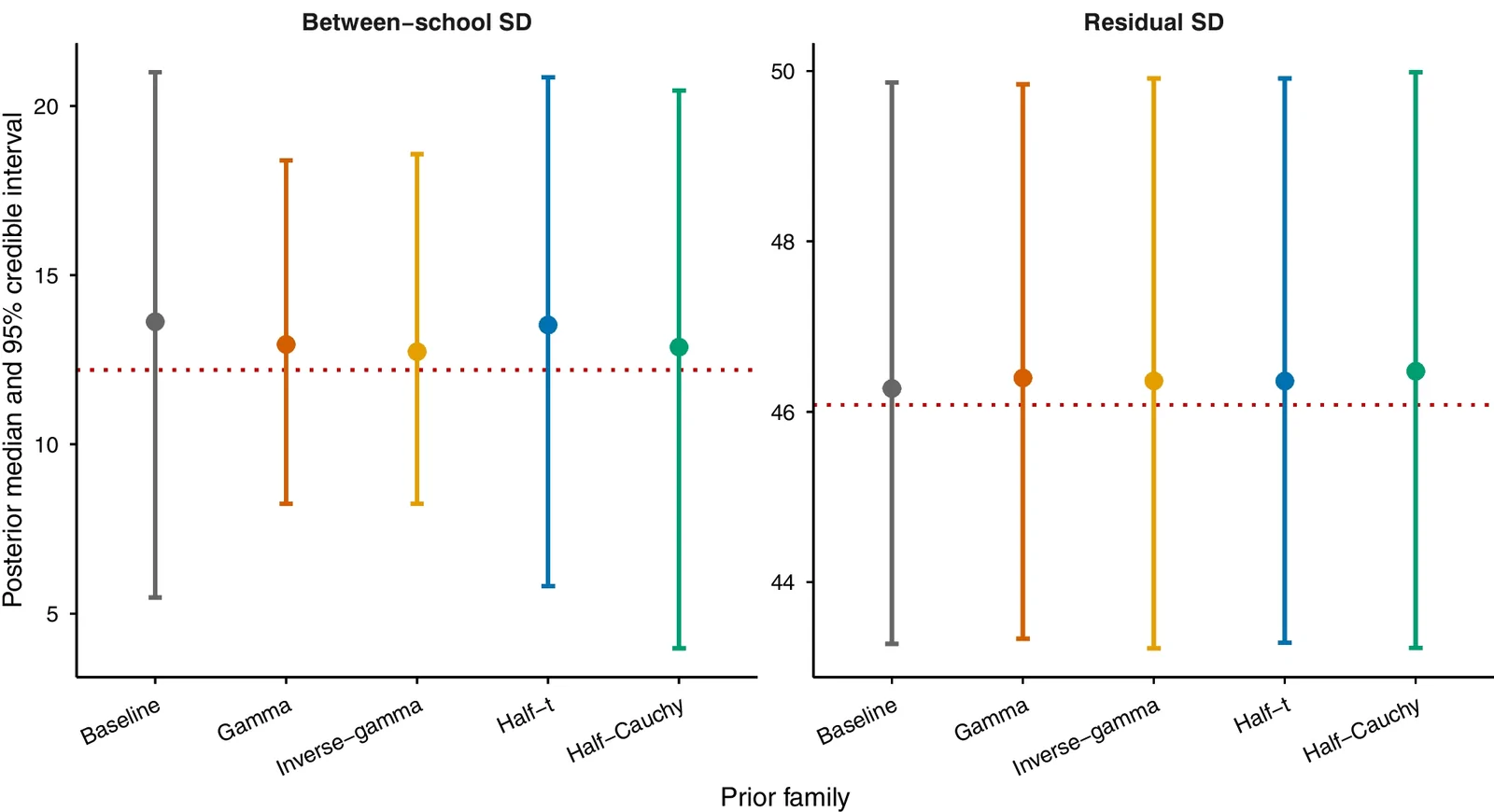
Posterior summaries for variance components across prior conditions in the TIMSS worked example. *Note.* Points represent posterior medians, and error bars represent equal-tailed 95% credible intervals. The left panel shows the between-school random-intercept standard deviation, and the right panel shows the residual standard deviation. Horizontal dotted lines indicate benchmark standard deviations from the non-overlapping reference sample. The worked example was based on a smaller sample of 40 schools with 10 students per school. Across conditions, the model specification and fixed-effect priors were held constant, while the variance-prior specifications differed. Baseline denotes a weakly informative reference specification using half-*t* priors with 3 degrees of freedom, location 0, and scale 20 on the standard-deviation scale

After fitting the worked example model, we compared posterior summaries for the two variance components across prior conditions. Posterior medians, equal-tailed 95% credible intervals, $\widehat{R}$, bulk effective sample size, and tail effective sample size for the focal variance components are reported in Table 5 and displayed in Fig. 4. Figure 4 is intended as a compact cross-condition summary of posterior location and interval width; it should be interpreted together with the prior-density comparison in Fig. 2, the prior predictive comparison in Fig. 3, and the supplementary posterior and diagnostic summaries. For the between-school random-intercept standard deviation, posterior medians ranged from approximately 12.74 under the inverse-gamma condition to 13.62 under the baseline condition. For the residual standard deviation, posterior medians ranged from approximately 46.27 to 46.48 across conditions. Thus, for both variance components, posterior location was fairly stable across priors. Interval widths differed modestly, particularly for the between-school component. The gamma and inverse-gamma conditions yielded somewhat tighter intervals, whereas the heavier-tailed half-*t* and half-Cauchy conditions produced somewhat wider intervals. This pattern is consistent with the prior-density comparison in Fig. 2 and shows that priors sharing a common benchmark location can still differ in the degree of posterior regularization they impose.

**Table 5:** Posterior summaries for variance components across prior conditions in the TIMSS worked example

Prior condition	Variance component	Posterior median	Equal-tailed 95% credible Interval	$\widehat{R}$	Bulk ESS	Tail ESS
Baseline	Between-school SD	13.62	[5.47, 21.00]	1.002	1,290.79	1,031.62
	Residual SD	46.27	[43.28, 49.86]	1.002	4,438.02	2,924.43
Gamma	Between-school SD	12.95	[8.25, 18.39]	0.999	2,386.12	2,675.20
	Residual SD	46.40	[43.33, 49.85]	1.001	4,741.37	2,923.38
Inverse-gamma	Between-school SD	12.74	[8.24, 18.57]	1.001	2,407.14	2,894.35
	Residual SD	46.36	[43.22, 49.91]	1.000	5,367.85	3,220.92
Half-*t*	Between-school SD	13.53	[5.81, 20.84]	1.003	1,092.13	987.37
	Residual SD	46.36	[43.29, 49.91]	1.001	4,079.29	3,136.21
Half-Cauchy	Between-school SD	12.87	[3.98, 20.46]	1.006	898.52	650.25
	Residual SD	46.48	[43.23, 49.99]	1.002	3,254.87	3,232.33

Posterior summaries are based on the worked example sample of 40 schools with 10 students per school. Values shown are posterior medians and equal-tailed 95% credible intervals for the between-school random-intercept standard deviation and the residual standard deviation under each prior condition. $\widehat{R}$ denotes the rank-normalized potential scale reduction statistic, and ESS denotes effective sample size. The baseline condition used a user-defined weakly informative half-*t*(3, 0, 20) specification on the standard-deviation scale and is included as a reference condition rather than as a benchmark-calibrated prior family

Although the present tutorial focuses on variance-prior construction, it is also informative to consider whether the fixed effects changed materially across prior conditions. In this worked example, the posterior summaries for the fixed effects were broadly stable across prior conditions (see Supplementary Table A). Because fixed-effect priors were held constant across these conditions, this pattern should not be interpreted as a general test of fixed-effect prior sensitivity. Rather, it indicates that, within the present comparison, changing the variance priors while holding the fixed-effect priors constant did not materially alter the fixed-effect summaries.

We also evaluated whether the fitted models behaved well computationally under the MCMC-based estimation used in this worked example. Sampler diagnostics are summarized in Table 6, which reports divergent transitions, maximum tree-depth hits, mean acceptance statistic, and mean step size across prior conditions. Across prior conditions, no divergent transitions or maximum tree-depth warnings were observed, and acceptance statistics were satisfactory. Convergence diagnostics for the variance components were likewise acceptable: $\widehat{R}$ values were close to 1.00, and effective sample sizes were adequate across conditions, although the half-Cauchy condition showed somewhat smaller effective sample sizes than the more concentrated specifications. Taken together, these diagnostics indicate that the fitted models were computationally stable and that the posterior comparisons were not driven by obvious sampling pathologies. Convergence assessment in the worked example was therefore based on multiple complementary diagnostics, including $\widehat{R}$, bulk effective sample size, tail effective sample size, divergent transitions, maximum tree-depth hits, and mean acceptance statistics. The adequacy of the posterior sample was therefore evaluated from the combined convergence and sampler diagnostics. Representative trace plots and autocorrelation plots for the key variance-component parameters across the main prior conditions are provided in Supplementary Figs. C and D. Supplementary posterior density plots for the focal variance components across prior conditions are provided in Supplementary Fig. E.

**Table 6:** Sampler diagnostics across prior conditions in the TIMSS worked example

Prior condition	Divergent transitions	Maximum tree-depth hits	Mean acceptance statistic	Mean step size
Baseline	0	0	0.9859	0.1380
Gamma	0	0	0.9902	0.1263
Inverse-gamma	0	0	0.9907	0.1271
Half-*t*	0	0	0.9878	0.1287
Half-Cauchy	0	0	0.9877	0.1241

Diagnostics are based on the fitted models for the worked example sample. “Maximum tree-depth hits” refers to the number of transitions that reached the specified maximum tree depth. Across all prior conditions, no divergent transitions or maximum tree-depth warnings were observed

Because benchmark-informed priors necessarily involve analyst choices about concentration and tail behavior, we also examined the robustness of the main findings through sensitivity analyses. Specifically, sensitivity analyses held the benchmark location fixed while varying prior concentration within the gamma family and tail behavior within the half-*t* family (see Supplementary Table B). Across these alternative settings, posterior summaries for the residual standard deviation were highly stable, and posterior summaries for the between-school standard deviation varied only modestly. In other words, although the exact interval widths and degree of shrinkage changed somewhat across alternative calibrations, the substantive pattern of results was unchanged. Convergence and sampler diagnostics were also satisfactory across sensitivity conditions (see Supplementary Tables B1 and B2). These results suggest that the benchmark-informed workflow can yield interpretable and stable inferences without depending on a single arbitrary hyperparameter choice.

Finally, this worked example illustrates the reporting principles that accompany prior construction in applied work. At a minimum, researchers should report the benchmark values used for calibration, the parameter scale on which the priors were specified, which hyperparameters were fixed and which were solved for, the rationale for the chosen level of prior concentration or tail behavior, prior predictive evidence showing what the priors imply on the observed-data scale, and sensitivity and diagnostic results demonstrating that the conclusions are not driven by a single unexamined specification. Thus, the TIMSS illustration shows that careful prior construction is not only a matter of choosing a prior family, but also of checking what that choice implies, evaluating whether inferences are robust to reasonable alternatives, and documenting those decisions clearly enough for readers to assess their substantive and methodological consequences.

Together, these five steps illustrate how benchmark-informed prior construction can be implemented as a transparent workflow that links substantive plausibility, mathematical calibration, software compatibility, and empirical evaluation.

## Discussion

This tutorial presented a benchmark-informed workflow for specifying and evaluating variance priors in Bayesian multilevel models. The workflow organizes prior construction as a sequence of linked decisions: identifying the target variance components and implementation scale, selecting benchmark values, expressing those benchmarks conceptually on the variance scale, calibrating software-compatible priors on the standard-deviation scale, and evaluating their implications through prior inspection, prior predictive checks, posterior comparison, and sensitivity analysis (Gabry et al., 2019; Schad et al., 2021). Using TIMSS 2019 as a worked example, the tutorial illustrated how this workflow can be implemented transparently in a familiar applied setting.

A first contribution of the tutorial is the distinction it makes between benchmark location and prior informativeness. In the worked example, benchmark values were obtained from a non-overlapping reference sample and used to anchor the location of the prior specifications, whereas the concentration and tail behavior of the priors were chosen separately to yield an interpretable comparison in a smaller-sample multilevel setting. This separation is important in practice. A benchmark can provide a plausible magnitude for a variance component, but it does not by itself determine how concentrated the prior should be around that value. By making these decisions explicit, the tutorial clarifies how empirical information can inform prior location without implying that there is a single uniquely correct prior specification.

A second contribution is the distinction between conceptual calibration and software implementation. Step 3 showed how benchmark information can be expressed on the variance or precision scale using familiar analytical relationships, whereas Step 4 translated that same information into standard-deviation-scale priors suitable for estimation in brms. This distinction matters because many discussions of variance priors are framed on the variance scale, whereas software such as brms requires prior specification on the standard-deviation scale for group-level and residual scale parameters (Bürkner, 2017, 2018). The tutorial therefore bridges a common gap between general Bayesian theory and applied implementation by showing how the same benchmark information can be used in both contexts without treating the software-compatible priors as exact one-to-one transformations of the conceptual variance-scale forms.

The TIMSS worked example illustrates how the workflow can reveal differences among prior families even when they are aligned to a common benchmark location. The tutorial value of this comparison lies in showing that benchmark-matched prior families do not become interchangeable simply because they share a common location target. On the parameter scale, the gamma and inverse-gamma priors placed relatively more mass near the benchmark values, whereas the half-*t* and especially the half-Cauchy priors retained heavier tails. On the outcome scale, these differences translated into broader prior predictive dispersion under the heavier-tailed specifications, with the half-Cauchy prior showing the widest upper range. These patterns illustrate why prior-family choice is not only a mathematical issue. It also affects what the model considers plausible before observing the focal data. The TIMSS example therefore serves as an illustration of how the workflow operates: prior families aligned to the same benchmark can still imply different prior predictive behavior and different degrees of posterior regularization.

At the same time, posterior results in the worked example were relatively stable across prior conditions. For the between-school random-intercept standard deviation, posterior medians varied only modestly across the benchmark-informed prior families and the baseline reference condition. For the residual standard deviation, posterior medians were highly stable across conditions. The main differences appeared in interval width rather than posterior location: the gamma and inverse-gamma priors yielded somewhat tighter intervals for the between-school component, whereas the heavier-tailed half-*t* and half-Cauchy priors produced somewhat wider intervals. This pattern was most visible for the between-school random-intercept standard deviation, which is consistent with prior work showing that estimation of higher-level variance components becomes more difficult in multilevel models with a small number of clusters (Bolin et al., 2019; Maas & Hox, 2005; McNeish & Stapleton, 2016b). In this worked example, the choice of prior family mattered more for the degree of posterior regularization than for the basic substantive pattern of results. Because the TIMSS application does not provide known population values, these differences should not be interpreted as evidence that tighter intervals imply better performance in terms of bias, RMSE, or credible-interval coverage. Here, greater regularization should be understood as reducing implausible boundary behavior or excessive posterior dispersion when the likelihood is weak, whereas excessively concentrated priors can make posterior inferences too insensitive to the likelihood. When the benchmark location is misaligned with the target sample, this same regularization can also introduce bias by shrinking posterior variance estimates toward a value that is not substantively appropriate for the focal data. These considerations reinforce the importance of prior inspection, prior predictive checking, posterior comparison, and sensitivity analysis as connected parts of the workflow.

The fixed effects were also broadly stable across prior conditions. Because fixed-effect priors were held constant, this pattern should not be interpreted as a general test of fixed-effect prior sensitivity. Instead, it indicates that, under the present design, varying the variance priors primarily affected the targeted variance components while leaving the estimated regression coefficients comparatively stable. Together with the variance-component results, this suggests that benchmark-informed variance priors may meaningfully shape posterior uncertainty about variance components, even when fixed-effect summaries remain comparatively stable under the same design.

The diagnostic results further support this interpretation. Across prior conditions, the fitted models showed satisfactory convergence and sampler behavior, with no divergent transitions or maximum tree-depth warnings. Effective sample sizes were adequate overall, although the half-Cauchy condition showed somewhat smaller values than the more concentrated specifications. Sensitivity analyses led to a similar conclusion. When prior concentration was varied within the gamma family and tail behavior was varied within the half-*t* family, posterior summaries for the residual standard deviation remained highly stable and posterior summaries for the between-school standard deviation varied only modestly. These results suggest that the benchmark-informed workflow can yield interpretable and stable inferences without depending on a single arbitrary hyperparameter choice.

Taken together, the findings from the worked example suggest several practical considerations for applied researchers in education, psychology, and related fields. First, prior specification for variance components should be treated as a substantive modeling decision rather than as a default software option (Gelman, 2006). Second, when fully external prior information is unavailable, benchmark information from a related or separated source may still be used for provisional calibration, but it should not be treated as equivalent to independent prior knowledge and should be accompanied by explicit discussion of comparability, benchmark uncertainty, and sensitivity analyses. Third, the implementation scale matters. Researchers need to align their prior-construction logic with the parameterization required by the software they are using. Fourth, prior evaluation should not stop at listing hyperparameters. Prior densities, prior predictive implications, posterior summaries, diagnostics, and sensitivity analyses all contribute to transparent and credible Bayesian modeling practice (Gabry et al., 2019; Kruschke et al., 2021; Schad et al., 2021).

This reporting emphasis synthesizes existing work on variance priors, Bayesian workflow, and reporting standards rather than introducing de novo rules for prior selection (Gabry et al., 2019; Gelman, 2006; Kruschke et al., 2021; McNeish & Stapleton, 2016a). At a minimum, researchers should report the target variance components, the parameter scale on which priors were specified, the benchmark values used for calibration, which hyperparameters were fixed and which were solved for, the rationale for the chosen level of concentration or tail behavior, and the results of prior predictive and sensitivity analyses (Kruschke et al., 2021). Reporting these elements makes prior choice more transparent and allows readers to assess whether the analysis depends heavily on a single unexamined specification.

Several limitations should be noted. First, the present paper is a tutorial with one empirical worked example, not a general performance study of all variance-prior families across all multilevel settings. The TIMSS illustration shows how the workflow operates in one realistic application, but it does not establish universally optimal priors. Nor does it provide a formal comparison of bias, RMSE, or credible-interval coverage under known population values, as would be possible in simulation studies. Future simulation studies should compare alternative variance-prior families with respect to bias, RMSE, and credible-interval coverage under known population values and across a broader range of multilevel structures. Second, the benchmark values were drawn from a non-overlapping reference sample within the broader TIMSS 2019 dataset rather than from a wholly external study or expert elicitation exercise. This was appropriate for tutorial purposes, but future work could examine how the workflow behaves when benchmark information comes from genuinely external sources or from less certain prior knowledge. More broadly, the worked example conditions on benchmark values and calibration settings rather than propagating uncertainty in those quantities through hyperpriors. Accordingly, it should be understood as a benchmark-informed calibration strategy that fixes benchmark and concentration choices for tutorial purposes, rather than as a fully prespecified prior specification or a full hierarchical Bayes treatment of hyperparameter uncertainty. This choice was appropriate for a tutorial focused on transparent calibration, but it does not exhaust the range of coherent Bayesian strategies. In applications where benchmark information is uncertain, researchers may instead consider hierarchical specifications that place hyperpriors on calibration parameters, or alternative designs such as external elicitation, data splitting, or cross-validation to preserve clearer prior-likelihood separation.

Third, the worked example focused on a two-level random-intercept model. Future research should extend the same framework to more complex settings, including random-slope models, longitudinal multilevel models, generalized multilevel models, and latent-variable or measurement models. An important extension would be to multilevel models with covariance matrices of random effects, where prior specification must address not only individual variance components but also covariances among random effects. This also includes extensions in which predictors are decomposed using latent rather than manifest between-within formulations, so that prior specification must address not only scalar variance components but also the additional measurement and structural parameters introduced by such models. Fourth, although the sensitivity analyses strengthened the tutorial by varying concentration within the gamma family and tail behavior within the half-*t* family, future simulation studies could examine benchmark misalignment more systematically by varying both benchmark location and concentration under known population values.

Overall, this tutorial emphasizes a transparent workflow for constructing, implementing, checking, and reporting variance priors in Bayesian multilevel models. The TIMSS example shows that the central practical questions are where the prior is anchored, how concentrated it is, how it behaves under the software parameterization, and whether the resulting inferences are robust to reasonable alternatives. Framed this way, variance-prior specification becomes part of principled model building, with prior-density inspection, prior predictive checking, posterior comparison, sensitivity analysis, and transparent reporting serving as practical safeguards for applied Bayesian multilevel work.

## Supplementary Information

Below is the link to the electronic supplementary material.

Supplementary file1 (PDF 768 KB)

## Data Availability

The TIMSS 2019 data used in this tutorial are publicly available from TIMSS & PIRLS International Study Center at https://timssandpirls.bc.edu/timss2019/. Reproducibility code and supplementary materials are publicly available on OSF at https://osf.io/uvpt7/.

## Declarations

### Open practices statement

This tutorial involved secondary analysis of publicly available data. No original experiments were conducted, and the study was not preregistered. Reproducibility materials are described in the Data availability statement.
