# Supplementary material for: A tutorial on selecting, translating, and reporting variance priors in Bayesian multilevel models
Source: Behav Res Methods. 2026 Oct 19;58(11):328. doi: 10.3758/s13428-026-03201-y (PMC13680335; doi:10.3758/s13428-026-03201-y)
Supplement: Supplementary file 1 [file 13428_2026_3201_MOESM1_ESM.pdf]

Supplementary Material

A Tutorial on Selecting, Translating, and Reporting Variance Priors in Bayesian Multilevel Models

Supplementary Table A

Posterior Summaries for Fixed Effects Across Prior Conditions in the TIMSS Worked Example

Prior condition	Fixed effect	Posterior median	95% credible interval
Baseline	Intercept	523.33	[516.67, 529.45]
	Student-level math	57.60	[53.09, 62.13]
	School-level math	71.53	[65.14, 77.91]
	Cross-level interaction	-2.88	[-7.22, 1.72]
Gamma	Intercept	523.13	[517.09, 529.18]
	Student-level math	57.61	[53.03, 62.18]
	School-level math	71.63	[65.29, 77.79]
	Cross-level interaction	-2.77	[-7.47, 1.82]
Inverse-gamma	Intercept	523.26	[517.19, 529.14]
	Student-level math	57.60	[53.26, 62.03]
	School-level math	71.55	[65.30, 77.97]
	Cross-level interaction	-2.88	[-7.49, 1.93]
Half-*t*	Intercept	523.22	[516.95, 529.56]
	Student-level math	57.60	[52.93, 62.07]
	School-level math	71.54	[65.04, 77.91]
	Cross-level interaction	-2.86	[-7.72, 2.26]
Half-Cauchy	Intercept	523.19	[517.01, 529.36]
	Student-level math	57.59	[52.95, 62.26]
	School-level math	71.60	[65.57, 77.87]
	Cross-level interaction	-2.83	[-7.61, 2.01]

Note. Posterior summaries are based on the worked example sample of 40 schools with 10 students per school. The student-level math effect corresponds to the cluster-mean-centered mathematics predictor, the school-level math effect corresponds to the school-average mathematics predictor, and the cross-level interaction corresponds to their product term. Across prior conditions, fixed-effect posterior summaries were broadly stable, consistent with the variance-focused interpretation of the worked example.

Supplementary Table B

Sensitivity Analysis Results for Variance Components Across Alternative Calibration Settings in the TIMSS Worked Example

Sensitivity condition	Hyperparameter setting	Between-school SD, posterior median [95% CrI]	Residual SD, posterior median [95% CrI]
Gamma, weaker concentration	shape = 8 (L2), 20 (L1)	13.00 [7.75, 18.79]	46.39 [43.22, 49.96]
Gamma, main concentration	shape = 12 (L2), 30 (L1)	12.97 [8.41, 18.32]	46.32 [43.20, 49.76]
Gamma, stronger concentration	shape = 20 (L2), 45 (L1)	12.81 [8.76, 17.48]	46.34 [43.30, 49.80]
Half-*t*, heavier-tailed	df = 2	13.22 [5.39, 20.48]	46.40 [43.19, 50.09]
Half-*t*, main	df = 4	13.21 [5.09, 20.33]	46.44 [43.23, 50.14]
Half-*t*, lighter-tailed	df = 8	13.35 [5.71, 20.84]	46.40 [43.18, 50.00]

Note. Sensitivity analyses varied prior concentration within the Gamma family and tail behavior within the half-*t* family while holding benchmark location fixed. For Gamma priors, weaker, main, and stronger conditions corresponded to alternative shape settings on the standard-deviation scale for the between-school and residual components. For half-*t* priors, heavier-tailed, main, and lighter-tailed conditions corresponded to alternative degrees-of-freedom settings. Across these alternative calibrations, posterior summaries for the residual standard deviation were highly stable, and posterior summaries for the between-school standard deviation varied only modestly. CrI = credible interval.

Supplementary Table B1

Convergence Diagnostics for Sensitivity Analyses in the TIMSS Worked Example

Sensitivity condition	Variance component	$\hat{R}$	Bulk ESS	Tail ESS
Gamma, weaker concentration	Between-school SD	1.001	3656.44	4824.32
	Residual SD	1.001	8250.74	5588.49
Gamma, main concentration	Between-school SD	1.000	3927.62	5098.73
	Residual SD	1.000	8250.26	6195.73
Gamma, stronger concentration	Between-school SD	1.000	5733.64	5681.77
	Residual SD	1.000	9947.00	5646.00
Half-*t*, heavier-tailed	Between-school SD	1.002	2457.68	2163.09
	Residual SD	1.000	8655.39	5431.31
Half-*t*, main	Between-school SD	1.001	2271.30	1841.10
	Residual SD	1.000	6608.75	4980.90
Half-*t*, lighter-tailed	Between-school SD	1.000	2461.78	2807.08
	Residual SD	1.001	8293.09	5711.79

Note. $\hat{R}$ denotes the rank-normalized potential scale reduction statistic, and ESS denotes effective sample size. Across all sensitivity conditions, $\hat{R}$ values were close to 1.00 and effective sample sizes were adequate, indicating satisfactory convergence and mixing.

Supplementary Table B2

Sampler Diagnostics for Sensitivity Analyses in the TIMSS Worked Example

Sensitivity condition	Divergent transitions	Maximum tree-depth hits	Mean acceptance statistic	Mean step size
Gamma, weaker concentration	0	0	0.9927	0.1061
Gamma, main concentration	0	0	0.9934	0.1068
Gamma, stronger concentration	0	0	0.9924	0.1174
Half-*t*, heavier-tailed	0	0	0.9952	0.0767
Half-*t*, main	0	0	0.9921	0.0980
Half-*t*, lighter-tailed	0	0	0.9930	0.0937

Note. "Maximum tree-depth hits" refers to the number of transitions that reached the specified maximum tree depth. Across all sensitivity conditions, no divergent transitions or maximum tree-depth warnings were observed.

Supplementary Figure C

Trace Plots for Variance Components Across Prior Conditions in the TIMSS Worked Example

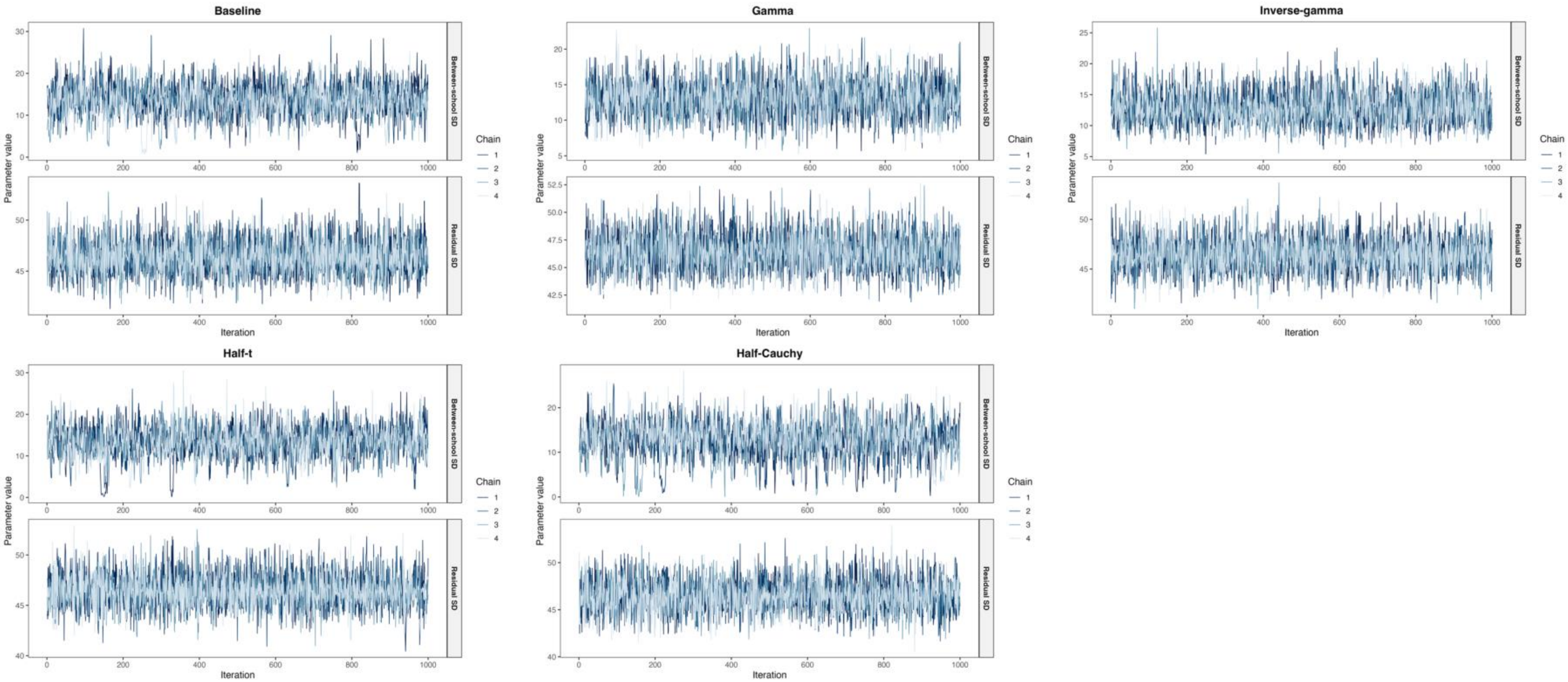

Note. Panels show trace plots for the between-school standard deviation and residual standard deviation under the Baseline, Gamma, Inverse-gamma, Half-*t*, and Half-Cauchy conditions. Across conditions, the chains overlapped well and showed no obvious signs of nonstationarity. Mixing was somewhat slower for the between-school standard deviation under the heavier-tailed conditions, consistent with the effective sample size summaries reported in the main text.

Supplementary Figure D

Autocorrelation Plots for Variance Components Across Prior Conditions in the TIMSS Worked Example

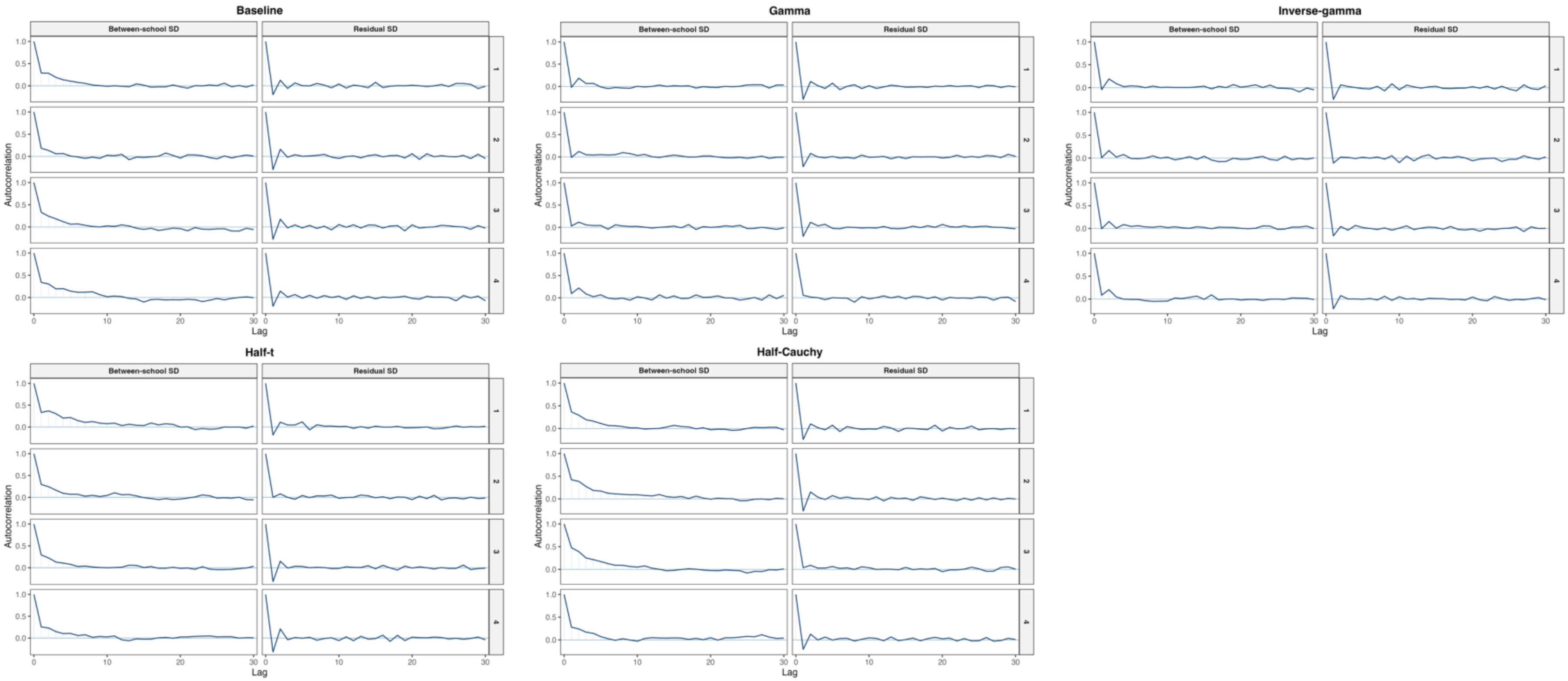

Note. Panels show autocorrelation plots for the same parameters and prior conditions as in Supplementary Figure C. Autocorrelation declined rapidly for the residual standard deviation across all conditions. For the between-school standard deviation, autocorrelation was somewhat higher at short lags under the heavier-tailed conditions, but it decreased toward zero with increasing lag, consistent with satisfactory overall mixing.

Supplementary Figure E

Posterior Density Plots for the Focal Variance Components Across Prior Conditions in the TIMSS Worked Example

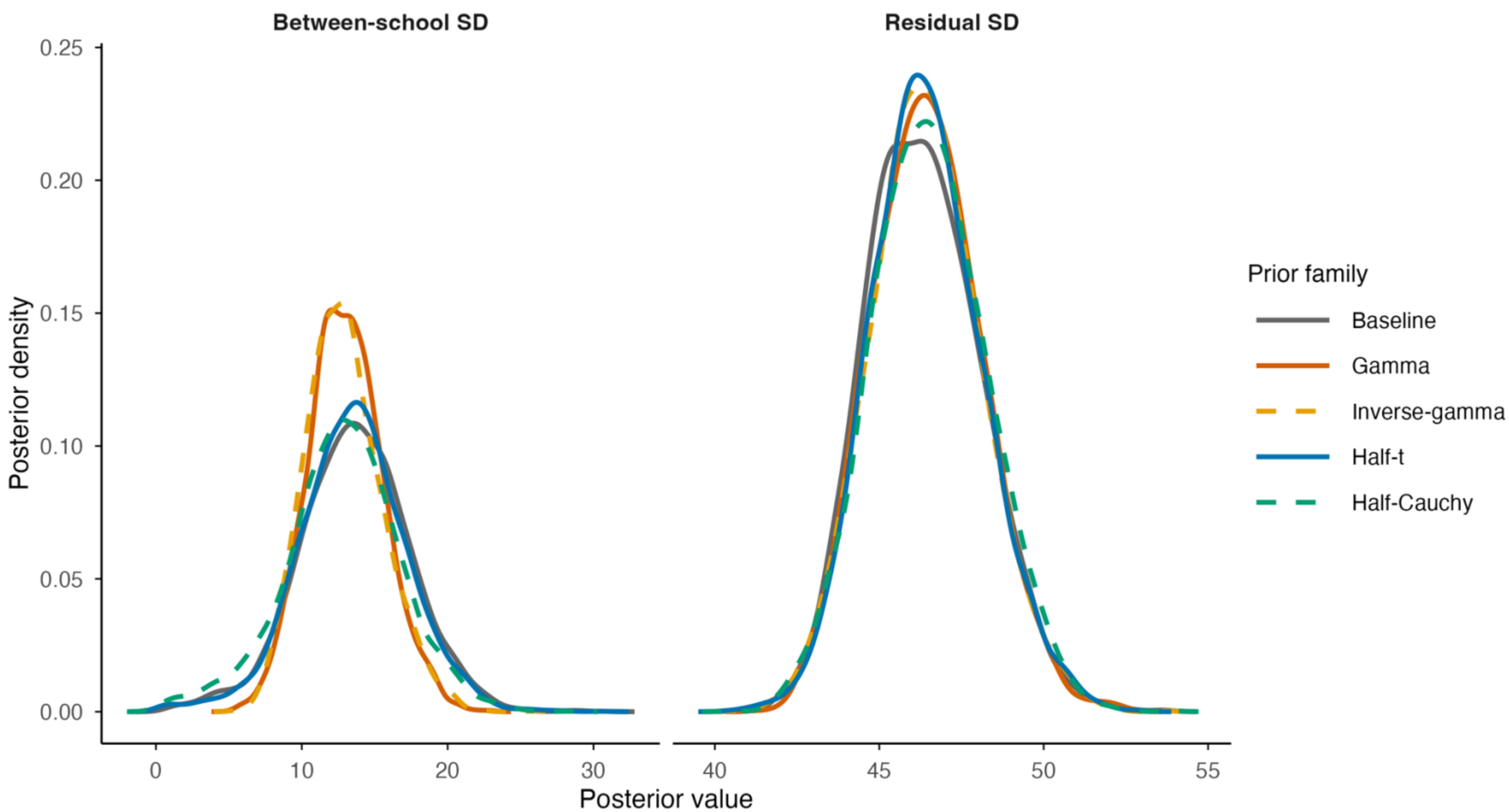

Note. The left panel shows posterior density estimates for the between-school random-intercept standard deviation, and the right panel shows posterior density estimates for the residual standard deviation. Curves are shown for the Baseline, Gamma, Inverse-gamma, Half-*t*, and Half-Cauchy prior conditions fit to the worked example sample. The figure is intended as a complementary visual display of posterior shape across prior conditions and should be interpreted together with the posterior medians and equal-tailed 95% credible intervals reported in Table 5 and displayed in Figure 4. Baseline denotes a weakly informative reference specification using half-*t* priors with 3 degrees of freedom, location 0, and scale 20 on the standard-deviation scale.
